# Association of dietary total antioxidant capacity and its distribution across three meals with all-cause, cancer, and non-cancer mortality among cancer survivors: the US National Health and Nutrition Examination Survey, 1999–2018

**DOI:** 10.3389/fnut.2023.1141380

**Published:** 2023-07-06

**Authors:** Peng Wang, Shengnan Zhao, Xiao Hu, Qilong Tan, Yaoyu Tan, Dan Shi

**Affiliations:** ^1^Department of Nutrition and Food Hygiene, School of Public Health, Chongqing Medical University, Chongqing, China; ^2^Department of Nutrition Food and Children’s Health, School of Public Health, Weifang Medical University, Weifang, China; ^3^Key Laboratory of Shaanxi Province for Craniofacial Precision Medicine Research, College of Stomatology, Xi’an Jiaotong University, Xi’an, China; ^4^Department of Epidemiology and Biostatistics, School of Public Health, Zhejiang University School of Medicine, Hangzhou, China; ^5^Research Center for Environment and Human Health, School of Public Health, Chongqing Medical University, Chongqing, China

**Keywords:** DAC, dinner, noncancer mortality, NHANES, nutrition

## Abstract

The effect of the antioxidant capacity of diet and its distribution across three meals on mortality risk among cancer patients remains unexplored. We aimed to prospectively investigate the association of dietary total antioxidant capacity (DAC) and its distribution across three meals with all-cause, cancer, and noncancer mortality among cancer survivors. We included 5,009 patients with cancer from the National Health and Nutrition Examination Survey conducted between 1999 and 2018. The adjusted hazard ratio (aHR) was estimated using the survey-weighted Cox proportional hazards model. During a median follow-up of 7.9 years, 1811 deaths, including 575 cancer-related deaths, were recorded. Among cancer survivors, compared with participants in the lowest quartile of total DAC from three meals, those in the highest quartile had a 24% decreased risk of noncancer mortality (aHR = 0.76, 95% confidence interval [CI]: 0.60–0.92), but not of all-cause and cancer mortality (each *p* trend >0.1). However, this association became insignificant for total DAC after excluding dinner DAC. In addition, higher dinner DAC rather than breakfast or lunch DAC was associated with a 21% lower risk of all-cause mortality (aHR = 0.79, 95% CI: 0.65–0.98) and 28% lower risk of noncancer mortality (aHR = 0.72, 95% CI: 0.57–0.90). Similar associations were found for ΔDAC (dinner DAC − breakfast DAC) with noncancer mortality (aHR = 0.56, 95% CI: 0.38–0.83), but DAC was not associated with cancer mortality (*p* trend >0.3). Among cancer survivors, total DAC from three meals was associated with reduced noncancer mortality, with the primary effect attributable to increased DAC intake from dinner. Our findings emphasize that DAC consumption from dinner should be advocated to reduce mortality risk in cancer survivors.

## 1. Introduction

There are approximately 33 million cancer survivors worldwide; this number is projected to increase due to population aging and improvements in the early detection and treatment of cancer (1). However, despite these advancements, people with cancer still have a shorter life expectancy than those without the disease (2). Cancer survival is defined as the time between cancer diagnosis and mortality (1, 3, 4). Diet is an important concern after cancer diagnosis for cancer survivors. Therefore, nutrition guidelines during and after cancer treatment have been introduced to improve the quality of life and mortality of patients with cancer, such as recommending the intake of abundant fruits and vegetables rich in antioxidant capacity (5–8). However, whether dietary antioxidants improve cancer survival is unknown.

Nevertheless, the association of dietary antioxidants with cancer mortality and prognosis has been widely reported in human and animal studies, but the findings have been largely diverse (9–15). Although some studies revealed a significant inverse association (10, 11, 13), others reported a null association (9, 12, 14), and a study reported the pro-tumorigenic role of dietary antioxidants (15). Emerging evidence has recently shown that in addition to the level and type of food, nutrition intake distribution across three meals can influence overall health (16–19). Therefore, we speculated that these inconsistent findings can be modified by monitoring dietary antioxidant distribution across three meals.

To date, only one study has investigated individual antioxidant distribution across three meals. It reported that antioxidants (i.e., vitamin C and E) in dinner were associated with lower cardiovascular disease (CVD) risks and all-cause mortality in the general population (20). However, whether dietary antioxidants across three meals would also impact mortality among cancer survivors was not clarified.

Given the importance of cumulative and/or synergistic effects of individual antioxidants from diets, we used an index of dietary total antioxidant capacity (DAC), a total estimate of the antioxidant capacity of all dietary antioxidants, to recapture an individual’s overall consumption of antioxidants in this study (21). We examined the associations of daily DAC distribution with the risk of all-cause, cancer, and noncancer mortality among cancer survivors in the US National Health and Nutrition Examination Survey (NHANES) cohort from 1999 to 2018.

## 2. Materials and methods

### 2.1. Study population

Participants were selected from the NHANES Cohort 1999–2018, a prospective study of health and nutrition established by the US National Center for Health Statistics (NCHS). For more details on the cohort, see https://www.cdc.gov/nchs/nhanes/index.htm. The project was approved by the NCHS review board, and all participants provided written informed consent before enrollment.

### 2.2. Inclusion/exclusion criteria

**Figure 1:**
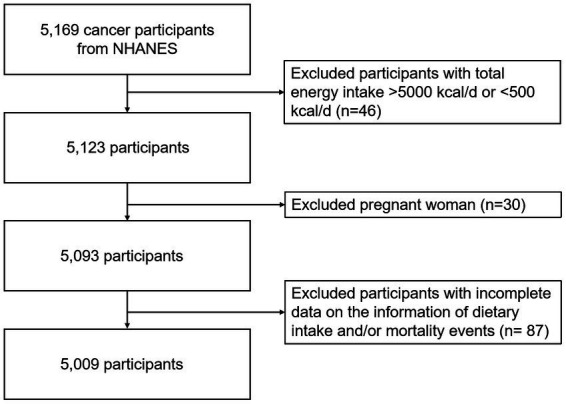
Cohort flow diagram of cancer survivors enrolled in NHANES.

The inclusion criteria in this analysis were that participants had at least one valid dietary recall interview and were diagnosed with cancer at baseline. We excluded participants who had total energy intake >5,000 kcal/d or < 500 kcal/d (*n* = 46), were pregnant (*n* = 30), and had missing dietary intake and/or mortality events (*n* = 87), leaving 5,009 participants for final analysis during a median follow-up of 7.9 years (1999–2018) (Figure 1).

### 2.3. Exposure assessment

Two non-consecutive 24-h dietary recalls were used to investigate the quantity, quality, and time of food intake; details have been described on the NHANES official website. Further, dietary supplement data were further collected by using a dietary supplement questionnaire. Strict, standardized protocols were performed to ensure the quantity of the interview quality. Nutrient compositions were acquired from the national nutrient database of the United States Department of Agriculture (USDA).

The antioxidant capacity assigned to each food item was expressed as the ferric-reducing ability of plasma (FRAP) value based on the single-electron transfer method. It was calculated with energy adjustment using the Antioxidant Food Database and the USDA’s Food Patterns Equivalents Database 2015–2016 (FPED 2015–2016) (22). The dietary antioxidant capacity per equivalent serving of the 30 categories of foods defined by the FPED 2015–2016 was presented (Supplementary Table S1). DAC was further calculated as the summation of the serving size of each food item multiplied by the FRAP value (mmol/serving) of each food item. The total DAC (the sum of three meals a day), DAC distribution (breakfast, lunch, and dinner), and the difference between dinner and breakfast (ΔDAC = dinner DAC − breakfast DAC) were further calculated. DAC from coffee and dietary supplements was excluded because of the inconclusive antioxidant ability of coffee with high content, which may weaken the correlation between DAC from other foods and mortality (23). The intake time of supplements was also missing.

### 2.4. Defining outcomes

All deaths (time and cause) were ascertained by linkage with the National Death Index through 31 December 2019. The outcome of interest was mortality: cancer mortality, defined as deaths due to cancer coded as the main cause of death (10th revision of International Classification of Disease, C00-C97), noncancer mortality, and all-cause mortality. The numbers of cases by cancer code in 5009 cancer cases are presented in Supplementary Table S2.

### 2.5. Statistical analysis

The analytic guidelines released by NHANES were adapted for all analyses incorporating complex sampling design methods of sample weights, stratification, and clustering. Descriptive statistics were used to summarize the baseline characteristics, expressed as mean or median with standard deviation (SD) or standard error (SE) for continuous variables and percentages for categorical variables. Data were weight-adjusted as appropriate. We performed one-way ANOVA for continuous variables and used the Chi-square test for categorical variables to examine baseline characteristics.

The survey-weighted Cox proportional hazards model is an officially recommended method in NHANES data^1^ and has been widely applied in previous studies (24, 25). Therefore, we also used survey-weighted Cox proportional hazards models to assess the associations of DAC (total, breakfast, lunch, and dinner; by quartiles) with all-cause, cancer, and noncancer mortality, showing adjusted hazard ratio (aHR) and 95% confidence interval (CI). To eliminate the difference between breakfast and dinner food types, we also evaluated the associations of ΔDAC (by quartiles) with all-cause, cancer, and noncancer mortality. Additionally, the linear or non-linear relationship between total and dinner DAC and mortality risk was analyzed using a restricted cubic spline model. Subgroup analysis was further performed, categorized by age (<60 or ≥ 60 years), sex (male or female), and body mass index (BMI) (<25, 25–30, or > 30 kg/m^2^) in survey-weighted Cox proportional hazards models.

Covariates were adjusted in three models. Model 1 was adjusted for age, sex (male; female), and race/ethnicity (Mexican American; non-Hispanic black; non-Hispanic white; other Hispanic; other). Model 2 was additionally adjusted for the following: education (below 9th grade; 9th–11th grade; college graduate or above; high school graduate/GED or equivalent; some college or associate of arts degree), family income ($ 0–$ 19,999; $20,000–$44,999; $45,000–$74,999; $75,000–$99,999; ≥$100,000), BMI, alcohol intake per day, dietary energy intake, smoking now or not, and physical activity per week (metabolic equivalent score, METs). Model 3 was further adjusted for serum high-density lipoprotein (HDL)-cholesterol, serum triglycerides, serum glycohemoglobin, diabetes, hypertension, CVD, dietary antioxidant supplement intake (vitamin C or vitamin E), and adherence to Healthy Eating Index 2015 (HEI-2015) score. Models for DAC intake at breakfast, lunch, and dinner were further adjusted, except the one defining the group. Diabetes was defined as having self-reported or diagnosed diabetes, hemoglobin A1c (HbA1c) ≥6.5%, or fasting plasma glucose ≥7.0 mmol/L. Hypertension was defined as diagnosed hypertension reported in NHANES. CVD was defined as diagnosed arthritis, congestive heart failure, coronary heart disease, angina, heart attack, or stroke.

To further validate the association between DAC from breakfast or dinner and mortality risk, we performed substitution analysis to partition one dietary item’s risk into another to calculate the relative risk for a fixed amount of intake (18, 26). In substitution analyses, we reassessed the associations of DAC with all-cause and noncancer mortality by replacing 10% DAC at breakfast with the equivalent amount of DAC or DAC from specific food at dinner.

In sensitivity analyses, we explored whether the associations persisted using the median value of DAC in survey-weighted Cox proportional hazards models. We further reassessed the weighted Cox proportional hazards models after including DAC from snack after dinner (food intake after 9 pm) data.

All statistical analyses were performed using R software (version 4.2.0). Missing data are described in Supplementary Table S3 and were imputed using multivariate imputation with chained equations. A two-sided *p*-value of <0.05 was deemed statistically significant.

## 3. Results

### 3.1. Baseline characteristics

During a median follow-up of 7.9 years, there were 5,009 cancer cases. At baseline, the mean age of participants was 61.67 years, and 52.3% were women. Among them, 39.9% consumed dietary antioxidant supplements. The average contents of total, breakfast, lunch, dinner, and snack after dinner DAC were 4.17 mmol, 1.11 mmol, 1.88 mmol, 1.18 mmol, and 0.31 mmol, respectively. Approximately 54.6, 24.5, and 58.1% of the participants had a history of hypertension, diabetes, and CVD, respectively (Table 1). Compared with participants in the lowest quartiles, those in higher total and dinner DAC quartiles were more likely to be non-Hispanic white, leaner, have a college graduate degree or above, and adhere to the Healthy Eating Index (HEI)-2015 score. In addition, they had higher physical activity and serum HDL-cholesterol levels but lower glycohemoglobin levels. They were less likely to be current smokers or have a history of CVD (Supplementary Tables S4, S5). Moreover, participants with higher ΔDAC consumption, relative to lowest consumption, were more likely to be women, be Mexican American, have a college or associate of arts degree, and have higher physical activity and higher serum HDL-cholesterol levels. In addition, they were less likely to be current smokers, adhere to HEI-2015 scores, have a history of CVD, and have a lower dietary antioxidant supplement intake (Supplementary Table S6).

**Table 1:** Baseline descriptive characteristics of 5,009 cancer survivors.

Characteristics	Mean ± SE or *n* (%)
*Patients, n*	5,009
*Age (years)*	61.67 ± 0.40
*Female*	2,622 (52.3)
*Race/ethnicity*	
Mexican American	292 (6.2)
Non-Hispanic Black	604 (12.9)
Non-Hispanic White	3,405 (72.5)
Other Hispanic	214 (4.6)
Other	179 (3.8)
*Education*	
Less than 9th grade	464 (9.3)
9th–11th grade	602 (12.0)
College graduate or above	1,317 (26.3)
High school graduate/GED or equivalent	1,141 (22.8)
Some college or Associate of Arts degree	1,481 (29.6)
*Income*	
$ 0–$ 19,999	1,080 (21.6)
$20,000–$44,999	1,513 (30.2)
$45,000–$74,999	909 (18.1)
$75,000–$99,999	639 (12.8)
$100,000 and over	868 (17.3)
*BMI (kg/m^2^)*	28.47 ± 0.14
*Alcohol intake (g/day)*	7.62 ± 0.44
*Smoke status*	
Never smoked	2,209 (44.1)
Past smoker	2049 (40.9)
Current smoker	751 (15.0)
*Physical activity (METs-h/week)*	6.91 ± 0.10
*Dietary energy intake (kcal)*	1940.63 ± 18.83
*Adherence to HEI-2015 score*	53.00 ± 0.34
*Dietary antioxidant supplement intake (yes)*	2000 (39.9)
*Total DAC intake (mmol)*	4.17 ± 0.06
*Breakfast DAC intake (mmol)*	1.11 ± 0.03
*Lunch DAC intake (mmol)*	1.88 ± 0.04
*Dinner DAC intake (mmol)*	1.18 ± 0.04
*DAC in snack after dinner (mmol)*	0.31 ± 0.04
*Serum HDL-Cholesterol (mg/dL)*	58.23 ± 0.47
*Serum triglycerides (mmol/L)*	5.84 ± 0.03
*Glycohemoglobin (%)*	2.25 ± 0.05
*Hypertension*	2,735 (54.6)
*Diabetes*	1,227 (24.5)
*CVD*	2,908 (58.1)

BMI, body mass index; HEI-2015, Healthy Eating Index 2015; DAC, dietary total antioxidant capacity; METs, metabolic equivalent score; CVD, cardiovascular disease. Continuous variables were adjusted for survey weights of NHANES. Categorical variables were unweighted.

### 3.2. DAC and its distribution across three meals and mortality risk

**Figure 2:**
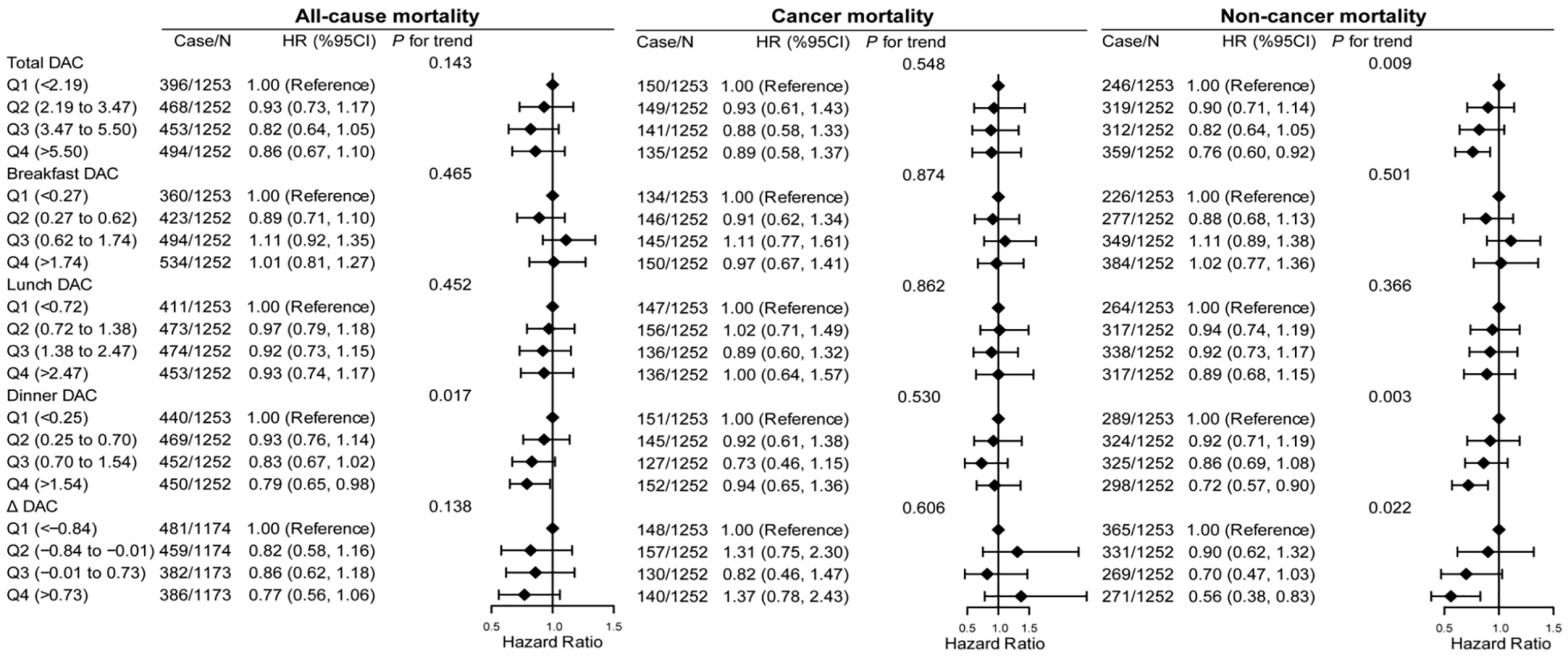
Association of all-cause, cancer, and noncancer mortality with DAC and its distribution across three meals in cancer survivors. HR, hazard ratio; CI, confidence intervals; DAC, dietary total antioxidant capacity; HEI-2015, Healthy Eating Index 2015; BMI, body mass index; METs, metabolic equivalent score; CVD, cardiovascular disease. *p for trend across the quartile of DAC. HR with 95% CI was assessed using weighted Cox regression analyses. Δ equals dinner DAC minus breakfast DAC. Models were adjusted for age, sex, race, education, family income, dietary energy intake, alcohol consumption per day, smoke status, METs, BMI, serum HDL-cholesterol, serum triglycerides, serum glycohemoglobin, diabetes, hypertension, CVD, dietary antioxidant supplement intake (vitamin C or vitamin E), and adherence to HEI-2015 score. Models for breakfast DAC intake, lunch DAC intake, and dinner DAC intake were further adjusted except the one that defined the group.

The associations of DAC and its distribution across three meals with mortality were evaluated using survey-weighted Cox proportional hazards models (Figure 2 and Supplementary Tables S7–S9). During the follow-up period, 1811 all-cause deaths occurred in the cohort, of which 575 were attributed to cancer and 1,236 to other noncancer causes. In adjusted Model 1 (age, sex, and race), a higher intake of total, breakfast, lunch, and dinner DACs and ΔDAC was progressively associated with lower all-cause and noncancer mortality risk (each *p* trend <0.05), with cancer mortality risk varying by distinct DAC groups. In Model 2, which was further adjusted for demographic and dietary factors, total DAC was still associated with lower all-cause and noncancer mortality (*P* trend <0.001 and *p* trend <0.001, respectively). However, there was no significant association between breakfast and lunch DACs and all-cause and noncancer mortality (*p* trend >0.1). The adjusted association with reduced all-cause and noncancer mortality risk for dinner DAC remained significant (*p* trend = 0.002 and *p* trend <0.001, respectively). For ΔDAC, the aHR for reduced noncancer mortality risk remained significant, while that for all-cause mortality became insignificant (all-cause mortality: *p* trend = 0.107; noncancer mortality: *p* trend = 0.029). However, further inclusion of disease and related markers in Model 3 attenuated these associations. Compared with the lowest quartiles, aHRs (95% CI) for all-cause and noncancer mortality associated with the highest quartiles of total DAC consumption were 0.86 (95% CI: 0.67–1.10) and 0.76 (95% CI: 0.60–0.92), respectively (all-cause mortality: *p* trend = 0.143; noncancer mortality: *p* trend = 0.009). However, compared with the highest quartiles of dinner DAC, they were 0.79 (95% CI: 0.65–0.98) and 0.72 (95% CI: 0.57–0.90), respectively (all-cause mortality: *p* trend =0.017; noncancer mortality: *p* trend = 0.003). Likewise, similar associations were found for ΔDAC with reduced all-cause mortality (all-cause mortality: 0.77 [95% CI: 0.56–1.06], *p* trend = 0.138; noncancer mortality: 0.56 (95% CI: 0.38–0.83), *p* trend = 0.022), but no significant associations existed between breakfast and lunch DACs and all-cause and noncancer mortality (each *p* trend >0.3). Notably, in multivariable-adjusted Models 2 and 3, total, breakfast, lunch, and dinner and ΔDACs were not associated with cancer mortality (each *p* trend >0.1).

Further, restricted cubic spline analysis showed a linear or non-linear relationship between total and dinner DAC and mortality risk (Supplementary Figure S1). There was non-linearity between total DAC and all-cause, cancer, and noncancer mortality. Meanwhile, dinner DAC and all-cause, cancer, and noncancer mortality had a linear inverse association.

Since noncancer mortality was inversely associated with dinner DAC, we further analyzed the associations of dinner DAC with the detailed causes of noncancer mortality (Supplementary Table S10). After adjusting for covariates, dinner DAC was associated with lower mortality risk due to chronic lower respiratory disease, nephritis, nephrotic syndrome and nephrosis, influenza and pneumonia, accidents (unintentional injuries), and heart diseases. Notably, a higher intake of dinner DAC was progressively associated with lower death due to Alzheimer’s disease and all other causes (each *p* trend <0.05). However, this association was not observed between dinner DAC and mortality due to diabetes mellitus and cerebrovascular diseases.

### 3.3. Associations of DAC stratified by food sources with mortality risk

**Figure 3:**
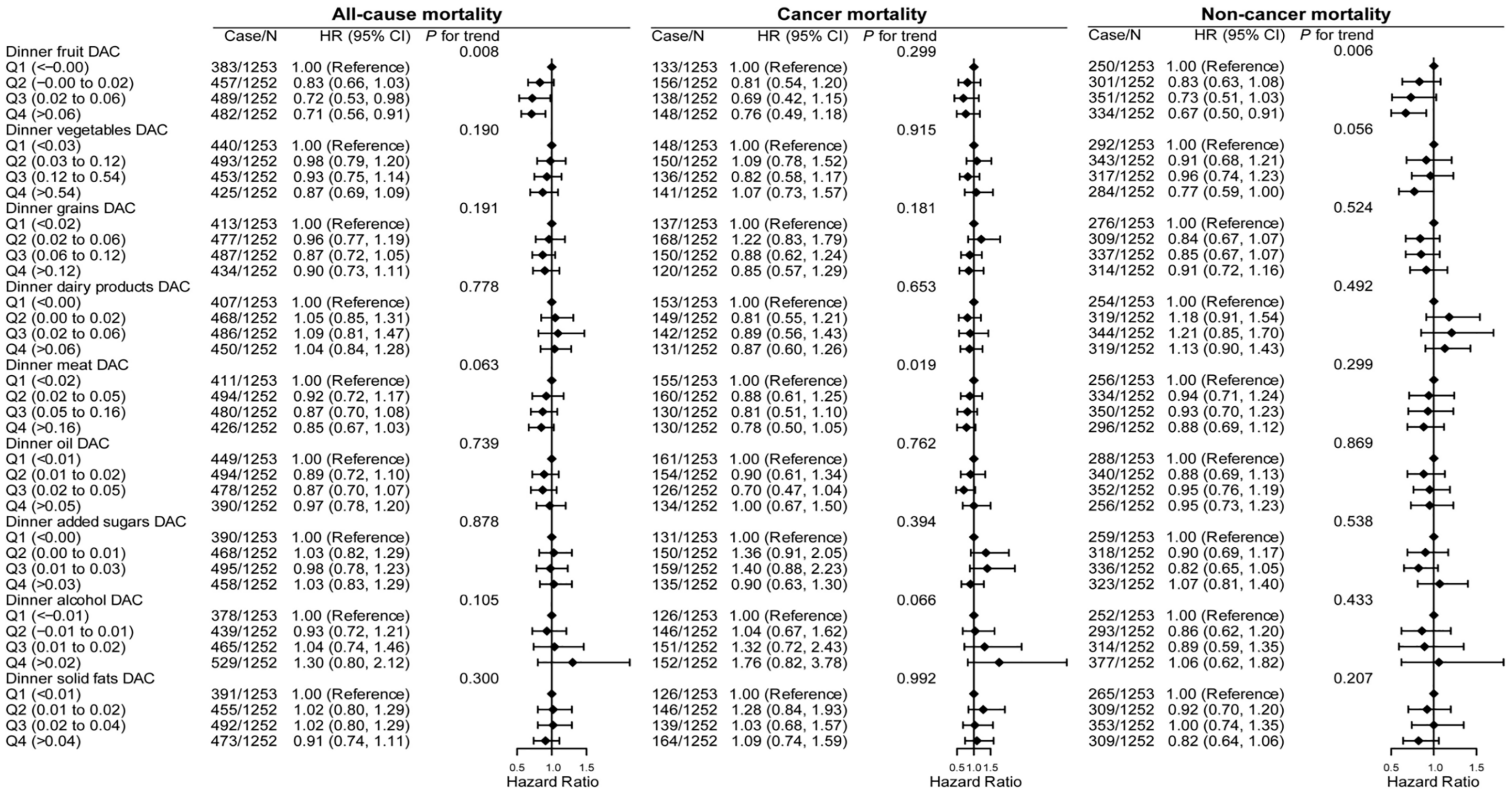
Association of all-cause, cancer, and noncancer mortality with dinner DAC, stratified by food sources in cancer survivors. HR, hazard ratio; CI, confidence intervals; DAC, dietary total antioxidant capacity; HEI-2015, Healthy Eating Index 2015; BMI, body mass index; METs, metabolic equivalent score; CVD, cardiovascular disease. *p for trend across the quartile of DAC. HR with 95% CI was assessed using weighted Cox regression analyses. Models were adjusted for age, sex, race, education, family income, dietary energy intake, alcohol consumption per day, smoke status, METs, BMI, serum HDL-Cholesterol, serum triglycerides, serum glycohemoglobin, diabetes, hypertension, CVD, dietary antioxidant supplement intake (vitamin C or vitamin E), adherence to HEI-2015 score, breakfast DAC intake, and lunch DAC intake.

Figure 3 and Supplementary Figure S2 show the associations of dinner and ΔDAC stratified by food sources with all-cause, cancer, and noncancer mortality. Compared with patients in the lowest quintile, those in the highest quintile of dinner fruit DAC had a lower risk of all-cause (aHR: 0.71, 95% CI: 0.56–0.91, *p* trend = 0.008) and noncancer mortality (aHR: 0.67, 95% CI: 0.50–0.91, *p* trend = 0.008). However, dinner DAC from other food sources, including vegetables, grains, dairy products, meat, oil, added sugars, alcohol, and solid fats, showed no association with all-cause, CVD, or cancer mortality. Likewise, ΔDAC stratified by food sources also showed no association with the risk of all-cause, CVD, and cancer mortality.

### 3.4. Subgroup analysis

Furthermore, subgroup analysis revealed that age, sex, and BMI did not impact the association between dinner DAC with noncancer mortality (Supplementary Table S11). A reverse association between dinner DAC with noncancer mortality was significantly observed only in females and persons with BMI >30 kg/m^2^. However, higher dinner DAC was still related to a lower risk trend of noncancer mortality in males, persons with BMI ≤30.0 kg/m^2^, and those aged <60 or ≥ 60 years.

### 3.5. Substitution analysis

**Figure 4:**
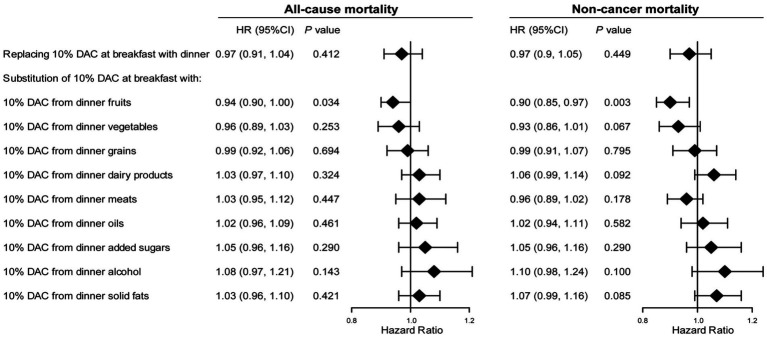
Substitution of DAC at breakfast with dinner and its association with all-cause and noncancer mortality in cancer survivors. HR, hazard ratio; CI, confidence intervals; DAC, dietary total antioxidant capacity; HEI-2015, Healthy Eating Index 2015; BMI, body mass index; METs, metabolic equivalent score; CVD, cardiovascular disease. *p for trend across the quartile of DAC. HR with 95% CI was assessed using weighted Cox regression analyses. Models were adjusted for age, sex, race, education, family income, dietary energy intake, alcohol consumption per day, smoke status, METs, BMI, serum HDL-Cholesterol, serum triglycerides, serum glycohemoglobin, diabetes, hypertension, CVD, dietary antioxidant supplement intake (vitamin C or vitamin E), adherence to HEI-2015 score, and lunch DAC (for substitution model of DAC at breakfast with dinner).

Figure 4 shows the reassessed association between DAC consumption and the risk of all-cause and noncancer mortality after replacing DAC consumption at breakfast with dinner. A hypothetical replacement of 10% DAC intake at breakfast with an equivalent proportion at lunch was not significantly associated with a lower risk of all-cause and noncancer mortality (each *p* > 0.1). Likewise, all-cause and noncancer mortality did not significantly decrease in models substituting 10% breakfast DAC with 10% dinner DAC from different foods, including vegetables, grains, dairy products, meats, oils, added sugars, alcohol, and solid fats (each *p* > 0.05). However, the replacement of 10% DAC intake at breakfast with an equivalent proportion of dinner DAC merely from fruits was associated with a decrease of 6% in all-cause mortality risk (aHR = 0.94, 95% CI: 0.90–1.00) and 10% in noncancer mortality risk (aHR = 0.90, 95% CI: 0.85–0.97).

### 3.6. Sensitivity analyses

In sensitivity analyses, the inverse association between total DAC and noncancer mortality became insignificant when we excluded dinner DAC from total DAC. No significant associations existed between total DAC and all-cause and cancer mortality (Supplementary Table S12). Further, the additional inclusion of DAC from snack after dinner to total or dinner DAC partially influenced the results; however, the trend was unchanged. Compared with the lowest quartiles, the total DAC obtained from including snacks after dinner showed aHRs for all-cause, cancer, and noncancer mortality of 0.88 (95% CI: 0.70–1.09), 0.95 (95% CI: 0.64–1.40), and 0.82 (95% CI: 0.63–0.1.06), respectively. Similarly, in the dinner DAC, including snacks after dinner group, the aHRs for all-cause, cancer, and noncancer mortality were 0.91 (95% CI: 0.75–1.10), 1.04 (95% CI: 0.76–1.43), and 0.84 (95% CI: 0.66–1.07), respectively. Therefore, DAC from snacks after dinner showed no association with all-cause, cancer, or noncancer mortality risks (Supplementary Table S13).

## 4. Discussion

To the best of our knowledge, this is the first study to investigate the association between DAC and its daily distribution and all-cause, cancer, and noncancer mortality in cancer survivors. We observed that among participants consuming high amounts of DAC from all three meals, the noncancer mortality risk decreased by 24%. In addition, among participants consuming DAC from dinner rather than breakfast or lunch, all-cause, and noncancer mortality risks robustly decreased by 21 and 28%, respectively. Furthermore, a higher ΔDAC was associated with a lower risk of noncancer mortality. This association was independent of multiple traditional risk factors, such as age, sex, BMI, and dietary and lifestyle factors. However, no association was observed between daily DAC intake distribution and cancer mortality in patients with cancer.

The relationship between individual dietary antioxidants and cancer death and incidence has been extensively explored with partially conflicting results (27–29). However, a single antioxidant may not reflect an individual’s overall consumption of antioxidants; therefore, we considered measuring the DAC to fill this gap. Many observational studies have investigated the association between DAC and the risk of cancer and mortality in the general population and yielded mixed findings (30–33). Some studies have revealed an inverse association (32, 33), while others have reported a null relationship (30, 31). Moreover, our prior findings showed that total DAC was negatively correlated with all-cause mortality in the general population but not with cancer and CVD mortality (unpublished data). Although cancer mortality has declined, the absolute mortality of patients with cancer has been increasing due to the high cancer incidence (1, 34). Thus, studies targeting cancer survivors have important public and clinical implications for improved cancer treatment. To date, only one study has investigated the association between DAC and cancer mortality among breast cancer survivors. The study found that total DAC was not associated with total mortality among breast cancer survivors (10). Following the prior study, our results showed that among people with cancer, no association existed between total DAC and all-cause or cancer mortality; however, total DAC was negatively associated with noncancer mortality.

More importantly, the findings of this present study are consistent with those of previous studies showing that a higher consumption of DAC from dinner than from breakfast was linearly associated with a lower risk of noncancer mortality in cancer survivors. Notably, we found that the inverse association between dinner DAC (but not total DAC) and mortality was attenuated if we included DAC intake from the snacks after dinner. This result suggests that if the meal timing is disrupted, the health benefit may be instantly compromised; thus, strict adherence to meal timing is needed in chrono-nutrition intervention strategies, as previously reported (35, 36). Moreover, this finding highlights the importance of meal timing of DAC on noncancer mortality risk in cancer survivors. Consistent with our findings, a previous study showed that dietary antioxidants, vitamin C, and vitamin E intake at dinner rather than at breakfast were related to reduced CVD and all-cause mortality in the general population (20). Although data supporting an association between meal timing of DAC and mortality in cancer survivors are scarce, other extensive studies that have examined the relationship between meal timing of nutrients and mortality similarly suggest the vital role of chrono-nutrition for survival. Previous bulk studies have shown that the optimal timing of meals differs when different nutrients are considered; some nutrients are best consumed at breakfast, whereas others are at dinner (16, 18, 37, 38). Further, studies have shown that dietary potassium, calcium, and magnesium intake at dinner was significantly associated with a lower risk of all-cause and cancer mortality (38). Meanwhile, a higher intake of energy, fat, and protein at dinner rather than breakfast increased all-cause, diabetes, and CVD mortality among people with diabetes (18).

Additionally, we found that DAC exclusively from dinner fruits significantly lowered the risk of all-cause and noncancer mortality. Generally, a healthy diet containing high vegetables and fruits plays a vital role in primary cancer prevention, but specific dietary recommendations for cancer survivors are lacking (39). Notably, a previous study reported that a high intake of prediagnosed fruit was associated with a lower risk of all-cause mortality in patients with ovarian cancer (39). However, whether fruit consumption reduces mortality risk in cancer survivors needs to be further investigated in other population studies. Furthermore, we found that ΔDAC stratified by food source was not associated with the risk of all-cause and cancer mortality. Therefore, this result suggests a minor effect of individual antioxidants; thus, combining individual antioxidants from different foods is required.

It is also worth noting that noncancer mortality (particularly death due to Alzheimer’s disease and all other causes), not cancer mortality, robustly reduced in association with total and dinner and ΔDACs among cancer survivors in this study. Notably, the three highest proportions of cancers recorded, i.e., skin cancer (nonmelanoma), breast cancer, and prostate cancer, accounting for 46% of patients with cancer in this study. Nonmelanoma skin cancer has a very low fatality rate, and its mortality tends to be related to poorer survival from causes unrelated to cancer in the affected patients (40–42). In addition, patients with breast and prostate cancers are also less likely to die of cancer but are more likely to die of noncancer causes, such as heart disease, infection, and suicide (43, 44). Thus, it is reasonable to conclude that targeting the lowering of noncancer mortality by increasing the consumption of total and dinner DACs is a promising strategy for survival improvement in patients with cancer. In particular, we considered that high consumption of total and dinner DACs might be more effective in lowering mortality risk among people with skin (nonmelanoma), breast, and prostate cancers; however, more research is warranted to confirm this suggestion.

The reasons for the association between total DAC, particularly high DAC from dinner rather than from breakfast, and reduced mortality in patients with cancer are complex. However, several biological processes may partially be used to explain this association. For instance, heightened oxidative and inflammatory stresses are commonly observed in cancer (45). Although some recent studies have demonstrated the anti-tumorigenic role of reactive oxygen species (ROS), others have shown that antioxidants possibly act in a pro-tumorigenic manner (15, 46). Nevertheless, many studies have recently suggested that antioxidants protect tumor cells from ROS- and DNA-induced damage that could lead to the proliferation of tumor cells (47, 48). Additionally, antioxidants play an important role as anti-inflammatory factors in the tumor process (49–52).

Most importantly, an intertwined relationship between the circadian rhythm and cancer has been extensively addressed, and targeting the circadian rhythm has been shown to inhibit cancer progression (53, 54). In particular, time-restricted feeding/eating has been shown to have a tumor suppression effect by synchronizing it with the circadian rhythm (55). Likewise, synchronizing dinner DAC with the rhythm may be related to reduced mortality, as exemplified by tumor necrosis factor-alpha (TNF-α) and interleukin-1β mRNA, with a night-time or afternoon peak rhythm (56, 57), consistent with the high consumption of dinner DAC. Moreover, this is further supported by the fact that the circadian clock regulates these inflammatory factors, which respond to meal timing (58). However, further studies are required to clarify these underlying mechanisms.

This study has several strengths. First, this is the first study to examine the association of daily DAC distribution with mortality in cancer survivors. Second, multivariable adjustment and a set of sensitivity analyses facilitated the representation of the association reported in this study. Third, this study used high-quality data with long-term follow-up from a well-designed cohort of the NHANES, which provided great statistical precision to assess the associations between DAC and the risk of mortality in cancer survivors.

However, this study has some potential limitations. First, although the repeatability and effectiveness of the dietary interviews were validated, long-term eating habits should be considered. Second, the external validity of our findings is lacking due to the missing information regarding meal timing and food intake in many datasets. Third, cooking methods that may alter the nutrient content and affect the antioxidant content of food are also lacking. Fourth, detailed information on cancer type, stage, or treatment is lacking, which would probably affect the association of DAC with cancer and noncancer mortality. Fifth, with the inconclusive antioxidant ability of coffee, excluding DAC from coffee and dietary supplements in this study may have weakened the association of DAC with mortality in cancer survivors. Finally, although traditional risk factors were comprehensively adjusted, we could not completely exclude the effect of unmeasured confounding factors. However, we believe that the findings of this study will facilitate evidence-based nutrient guidelines for cancer-directed care and improve survival and quality of life in patients with cancer.

In conclusion, among cancer survivors, the consumption of total DAC was inversely associated with the risk of noncancer mortality. This benefit was completely recaptured by higher DAC from dinner rather than breakfast or lunch, showing that a higher intake of dinner DAC was more favorable to the lower risk of all-cause noncancer mortality. High DAC consumption from dinner might be advocated for and incorporated into a healthy dietary pattern to reduce mortality risk in cancer survivors.

## Data availability statement

Publicly available datasets were analyzed in this study. This data can be found at: The data that support the findings of this study are available in [National Health and Nutrition Examination Survey] at [https://wwwn.cdc.gov/nchs/nhanes/default.aspx], [Food Patterns Equivalents Database] at [https://www.ars.usda.gov/northeast-area/beltsville-md-bhnrc/beltsville-human-nutrition-research-center/food-surveys-research-group/docs/fped-databases/], [Mortality data] at [https://ftp.cdc.gov/pub/Health_Statistics/NCHS/datalinkage/linked_mortality/]. These data were derived from the following resources available in the public domain: [National Center for Health Statistics, https://www.cdc.gov/nchs/index.htm; Agricultural Research Service, https://www.ars.usda.gov/].

## Author contributions

PW: conceptualization and formal analysis. YT: data curation. QT: methodology. DS: project administration, resources, and supervision. PW and QT: software. XH: validation. SZ and DS: writing—original draft. PW and DS: writing—review & editing. All authors contributed to the article and approved the submitted version.

## Funding

This research was funded by the National Natural Science Foundation of China (No. 8210120502).

## Conflict of interest

The authors declare that the research was conducted in the absence of any commercial or financial relationships that could be construed as a potential conflict of interest.

## Publisher’s note

All claims expressed in this article are solely those of the authors and do not necessarily represent those of their affiliated organizations, or those of the publisher, the editors and the reviewers. Any product that may be evaluated in this article, or claim that may be made by its manufacturer, is not guaranteed or endorsed by the publisher.

## Footnotes

^1^ https://wwwn.cdc.gov/nchs/nhanes/tutorials/Weighting.aspx

## Abbreviations

aHR: Adjusted hazard ratio
CVD: Cardiovascular disease
DAC: Dietary total antioxidant capacity
HEI: Healthy Eating Index
NHANES: National Health and Nutrition Examination Survey
BMI: Body mass index
METs: Metabolic equivalent score
