# Supplementary material for: Association of dietary total antioxidant capacity and its distribution across three meals with all-cause, cancer, and non-cancer mortality among cancer survivors: the US National Health and Nutrition Examination Survey, 1999–2018
Source: Front Nutr. 2023 Jul 6;10:1141380. doi: 10.3389/fnut.2023.1141380 (PMC10359731; doi:10.3389/fnut.2023.1141380)
Supplement: Supplementary file 1 [file Data_Sheet_1.docx]

Supplementary Material

Association of Dietary Total Antioxidant Capacity and its Distribution Across Three Meals with All-Cause, Cancer, and Noncancer Mortality Among Cancer Survivors: The US National Health and Nutrition Examination Survey, 1999-2018

**Peng Wang^1^** **^†^, Shengnan Zhao^2^** **^†^, Xiao Hu^3^, Qilong Tan^4^, Yaoyu Tan^1^, Dan Shi^1, 5, *^**

*** Correspondence:** Dan Shi: [danshi@cqmu.edu.cn](mailto:danshi@cqmu.edu.cn)

# Supplementary Figures and Tables

## Supplementary Figures

**
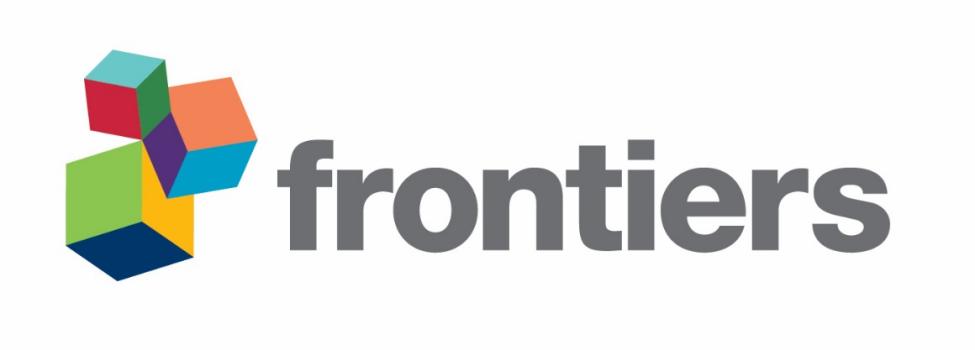
**


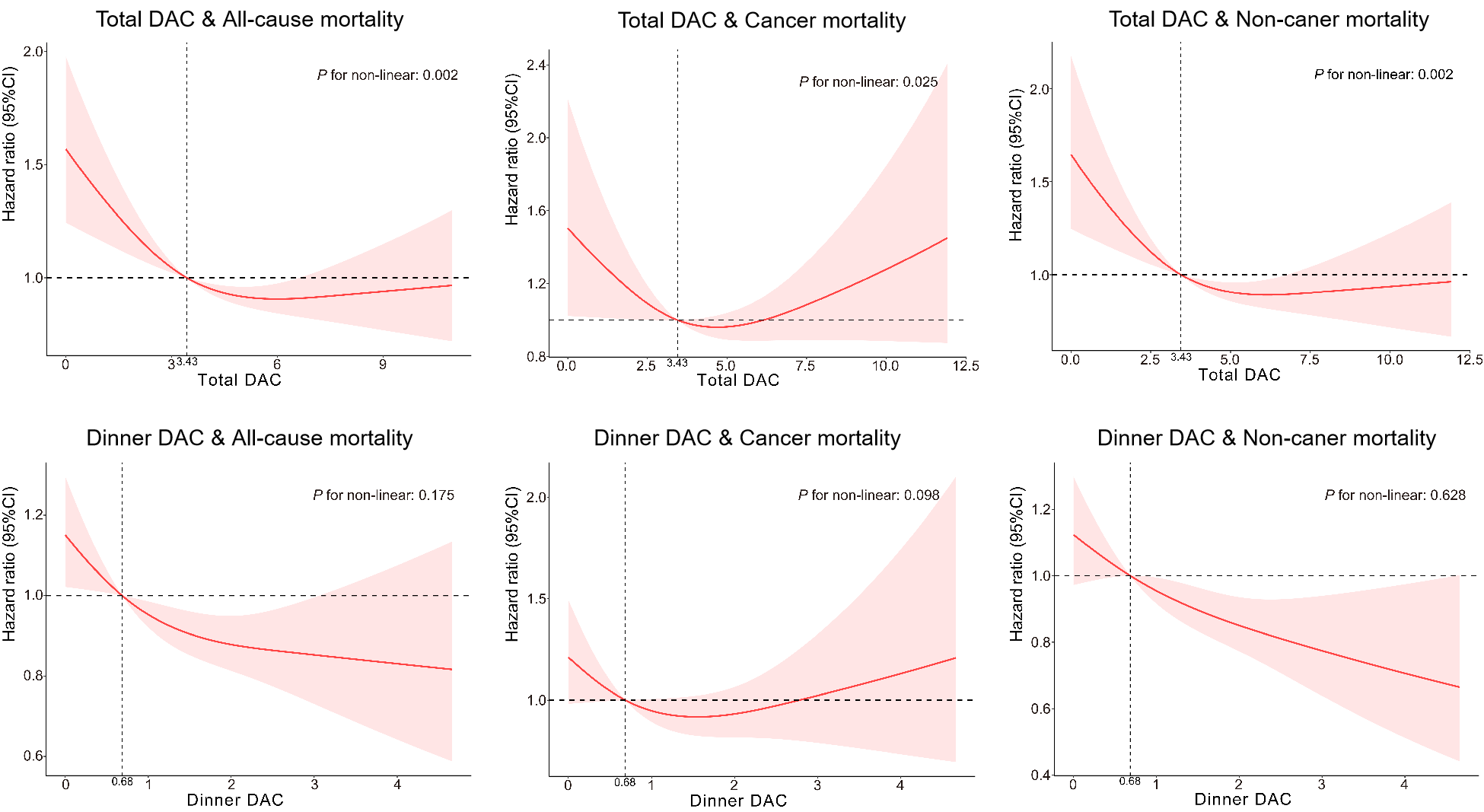


**Supplementary Figure 1.** HR of total and dinner DAC with all-cause, cancer and noncancer mortality using restricted cubic spline analysis. Abbreviations: HR, hazard ratio; DAC, dietary total antioxidant capacity; HEI-2015, Healthy Eating Index 2015; BMI, body mass index; METs, metabolic equivalent score; CVD, cardiovascular disease. Models were adjusted for age, sex, race, education, family income, dietary energy intake, alcohol consumption per day, smoke status, METs, BMI, serum HDL-cholesterol, serum triglycerides, serum glycohemoglobin, diabetes, hypertension, CVD, dietary antioxidant supplement intake (vitamin C or vitamin E), and adherence to HEI-2015 score. Models for dinner DAC were further adjusted for breakfast and lunch DAC.


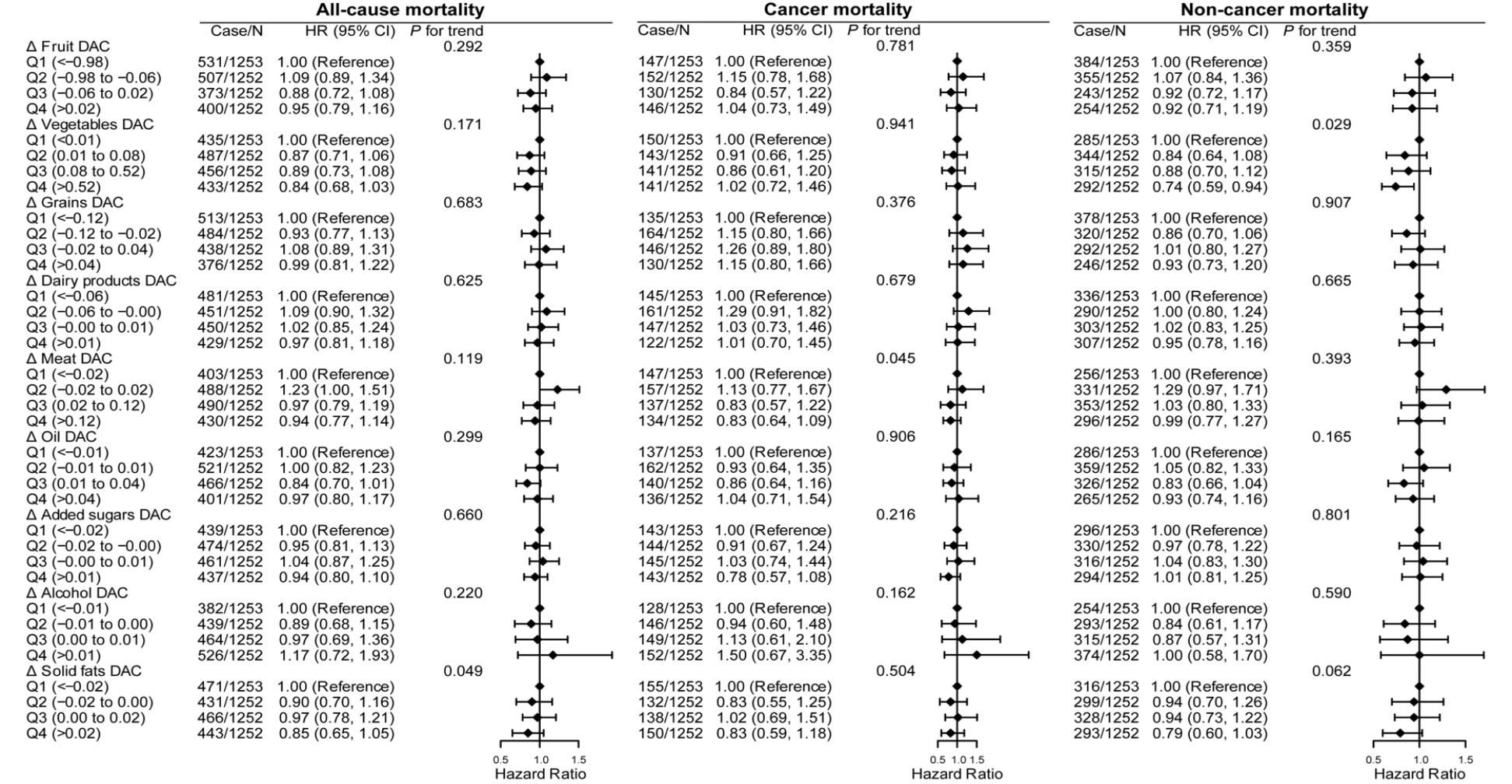


**Supplementary Figure 2.** Adjusted HRs for Δ DAC and all-cause mortality, cancer mortality and noncancer mortality, stratified by food sources in cancer survivors. Abbreviations: HR, hazard ratio; CI, confidence intervals; DAC, dietary total antioxidant capacity; HEI-2015, Healthy Eating Index 2015; BMI, body mass index; METs, metabolic equivalent score; CVD, cardiovascular disease. * *P* for trend across the quintile of DAC. HR (95%CI) was estimated using weighted Cox regression analyses. Δ equals dinner DAC from specific food minus breakfast DAC from the corresponding food. Models were adjusted for age, sex, race, education, family income, dietary energy intake, alcohol consumption per day, smoke status, METs, BMI, serum HDL-cholesterol, serum triglycerides, serum glycohemoglobin, diabetes, hypertension, CVD, dietary antioxidant supplement intake (vitamin C or vitamin E), and adherence to HEI-2015 score.

# Supplementary Tables

**Supplementary Table 1.** Mean antioxidant capacity per equivalent serving of the 30 categories of foods defined by the FPED

| **Main categories ^a^** | **FPID/FPED categories** | **Dietary antioxidant capacity (mmol/serving) ^b^** |
| --- | --- | --- |
|  |  |  |
| **Fruit** | Citrus, melons, and berries | 2.696 |
|  | Other fruits | 1.857 |
|  | Fruit juice | 1.182 |
|  |  |  |
| **Vegetables** | Dark green vegetables | 1.572 |
|  | Tomatoes | 0.806 |
|  | Other red and orange vegetables (excludes, tomatoes) | 0.330 |
|  | Potatoes (white potatoes) | 0.509 |
|  | Other starchy vegetables (excludes white potatoes) | 0.227 |
|  | Other vegetables | 1.182 |
|  | Beans and peas computed as vegetables | 0.561 |
|  |  |  |
| **Grains** | Whole grains | 0.173 |
|  | Refined grains | 0.043 |
|  |  |  |
| **Protein Foods** | Meat (beef, veal, pork, lamb, game) | 0.008 |
|  | Cured meat | 0.102 |
|  | Organ meat | 0.222 |
|  | Poultry | 0.049 |
|  | Seafood high in n-3 fatty acids | 0.031 |
|  | Seafood low in n-3 fatty acids | 0.031 |
|  | Eggs | 0.027 |
|  | Soybean products | 0.056 |
| (continued) |  |  |
|  | Nuts and seeds | 0.426 |
|  | Beans and peas computed as protein foods | 0.140 |
|  |  |  |
| **Dairy** | Milk (includes calcium fortified soy milk) | 0.148 |
|  | Yogurt | 0.098 |
|  | Cheese | 0.162 |
|  |  |  |
| **Oils** | Oils | 0.668 |
| **Solid Fats** | Solid fats | 0.339 |
| **Added Sugars** | Added sugars | 0.008 |
| **Alcoholic Drinks** | Alcoholic drinks | 1.326 |

^a^ The food classification was defined by of USDA's Food Patterns Equivalents Database 2015-2016 (FPED 2015-2016). b, the dietary antioxidant capacity value of the food components included in the different food categories defined by the FPED, respectively. The FPED dietary intake servings were calculated by energy adjustment.

**Supplementary Table 2.** Number of cases by cancer code among 5009 cancer cases

| **cancer code** | **cancer type** | **number** |  | **cancer code** | **cancer type** | **number** |
| --- | --- | --- | --- | --- | --- | --- |
| 10 | Bladder | 119 |  | 26 | Mouth/tongue/lip | 26 |
| 11 | Blood | 12 |  | 27 | Nervous system | 0 |
| 12 | Bone | 30 |  | 28 | Ovary (ovarian) | 109 |
| 13 | Brain | 23 |  | 29 | Pancreas (pancreatic) | 7 |
| 14 | Breast | 735 |  | 30 | Prostate | 736 |
| 15 | Cervix (cervical) | 354 |  | 31 | Rectum (rectal) | 21 |
| 16 | Colon | 325 |  | 32 | Skin (non-melanoma) | 812 |
| 17 | Esophagus (esophageal) | 25 |  | 33 | Skin (don't know what kind) | 384 |
| 18 | Gallbladder | 1 |  | 34 | Soft tissue (muscle or fat) | 10 |
| 19 | Kidney | 81 |  | 35 | Stomach | 34 |
| 20 | Larynx/ windpipe | 22 |  | 36 | Testis (testicular) | 38 |
| 21 | Leukemia | 44 |  | 37 | Thyroid | 101 |
| 22 | Liver | 20 |  | 38 | Uterus (uterine) | 192 |
| 23 | Lung | 117 |  | 39 | Other | 203 |
| 24 | Lymphoma/ Hodgkin's disease | 105 |  | 99 | Unidentified cancer | 31 |
| 25 | Melanoma | 292 |  |  |  |  |

**Supplementary Table 3.** Detailed information on missing data

| **Characteristic** | **Missing Count** | **Missing percent** |
| --- | --- | --- |
| **Age (years)** | 0 | 0.00% |
| **Female** | 0 | 0.00% |
| **Race/ethnicity** | 0 | 0.00% |
| **Education** | 4 | 0.10% |
| **Income** | 408 | 8.10% |
| **Body mass index (kg/m2)** | 112 | 2.20% |
| **Alcohol intake (g/day)** | 0 | 0.00% |
| **Smoke status** | 2 | 0.00% |
| **Physical activity (METs-h/week)** | 129 | 2.50% |
| **Dietary energy intake (kcal)** | 0 | 0.00% |
| **Adherence to HEI-2015 score** | 0 | 0.00% |
| **Dietary antioxidant supplement intake (yes)** | 568 | 11.30% |
| **Total DAC intake (mmol)** | 0 | 0.00% |
| **Breakfast DAC intake (mmol)** | 0 | 0.00% |
| **Lunch DAC intake (mmol)** | 0 | 0.00% |
| **Dinner DAC intake (mmol)** | 0 | 0.00% |
| **DAC in snack after dinner(mmol)** | 0 | 0.00% |
| **Serum HDL-Cholesterol (mg/dL)** | 602 | 12.00% |
| **Serum triglycerides (mmol/L)** | 367 | 7.30% |
| **Glycohemoglobin (%)** | 287 | 5.70% |
| **Hypertension** | 0 | 0.00% |
| **Diabetes** | 0 | 0.00% |
| **CVD** | 0 | 0.00% |

Abbreviations: BMI, body mass index; HEI-2015, Healthy Eating Index 2015; DAC, dietary total antioxidant capacity; METs, metabolic equivalent score; CVD, cardiovascular disease.

**Supplementary Table 4.** Baseline characteristics of cancer survivors by quartiles of total DAC

| **Characteristic** | **Total DAC** | | | | ***P* value** |
| --- | --- | --- | --- | --- | --- |
|  | **Q1** | **Q2** | **Q3** | **Q4** |  |
| **Patients, n** | 1253 | 1252 | 1252 | 1252 |  |
| **Age (years)** | 56.51±0.72 | 62.45±0.74 | 62.78±0.68 | 65.05±0.72 | <0.001 |
| **Female** | 660 (52.7) | 697 (55.7) | 670 (53.5) | 595 (47.5) | <0.001 |
| **Race/ethnicity** |  |  |  |  | <0.001 |
| Mexican American | 97 (7.7) | 96 (7.7) | 75 (6.0) | 52 (4.2) |  |
| Non-Hispanic Black | 226 (18.0) | 186 (14.9) | 132 (10.5) | 115 (9.2) |  |
| Non-Hispanic White | 824 (65.8) | 827 (66.1) | 937 (74.8) | 1008 (80.5) |  |
| Other Hispanic | 51 (4.1) | 83 (6.6) | 58 (4.6) | 39 (3.1) |  |
| Other | 55 (4.4) | 60 (4.8) | 50 (4.0) | 38 (3.0) |  |
| **Education** |  |  |  |  | <0.001 |
| Less than 9th grade | 122 (9.8) | 168 (13.4) | 99 (7.9) | 75 (6.0) |  |
| 9-11th grade | 186 (14.9) | 183 (14.6) | 139 (11.1) | 94 (7.5) |  |
| College graduate or above | 217 (17.4) | 236 (18.8) | 373 (29.8) | 491 (39.2) |  |
| High school graduate/GED or equivalent | 316 (25.3) | 291 (23.2) | 283 (22.6) | 251 (20.0) |  |
| Some college or Associate of Arts degree | 408 (32.7) | 374 (29.9) | 358 (28.6) | 341 (27.2) |  |
| **Income** |  |  |  |  | <0.001 |
| $ 0 to $ 19,999 | 311 (24.8) | 333 (26.6) | 252 (20.1) | 184 (14.7) |  |
| $20,000 to $44,999 | 409 (32.6) | 379 (30.3) | 377 (30.1) | 348 (27.8) |  |
| $45,000 to $74,999 | 215 (17.2) | 207 (16.5) | 232 (18.5) | 255 (20.4) |  |
| $75,000 to $99,999 | 122 (9.7) | 136 (10.9) | 168 (13.4) | 213 (17.0) |  |
| $100,000 and Over | 196 (15.6) | 197 (15.7) | 223 (17.8) | 252 (20.1) |  |
| **BMI (kg/m^2^)** | 28.39±0.29 | 29.11±0.30 | 28.61±0.28 | 27.84±0.28 | 0.010 |
| **Alcohol intake (g/day)** | 8.02±0.98 | 4.23±0.51 | 6.03±0.58 | 11.90±0.98 | <0.001 |
| **Smoke status** |  |  |  |  | <0.001 |
| Never smoked | 421 (33.6) | 574 (45.8) | 581 (46.4) | 633 (50.6) |  |
| Past smoker | 454 (36.2) | 503 (40.2) | 544 (43.5) | 548 (43.8) |  |
| (continued) |  |  |  |  |  |
| Current smoker | 378 (30.2) | 175 (14.0) | 127 (10.1) | 71 (5.7) |  |
| **Physical activity (METs-h/week)** | 6.69±0.18 | 6.30±0.18 | 7.07±0.18 | 7.51±0.26 | <0.001 |
| **Dietary energy intake (kcal)** | 2020.90±42.13 | 1787.27±28.01 | 1900.89±29.74 | 2039.38±33.28 | <0.001 |
| **Adherence to HEI-2015 score** | 43.24±0.47 | 49.54±0.59 | 55.46±0.45 | 63.49±0.58 | <0.001 |
| **Dietary antioxidant supplement intake (yes)** | 453 (36.2) | 509 (40.7) | 514 (41.1) | 524 (41.9) | 0.016 |
| **Serum HDL-Cholesterol (mg/dL)** | 52.95±0.88 | 55.69±0.87 | 59.91±1.05 | 64.15±1.02 | <0.001 |
| **Serum triglycerides (mmol/L)** | 5.80±0.05 | 5.99±0.05 | 5.85±0.04 | 5.74±0.04 | <0.001 |
| **Glycohemoglobin (%)** | 2.34±0.10 | 2.53±0.14 | 2.13±0.10 | 2.04±0.10 | 0.020 |
| **Hypertension** | 666 (53.2) | 742 (59.3) | 662 (52.9) | 665 (53.1) | 0.002 |
| **Diabetes** | 295 (23.5) | 359 (28.7) | 313 (25.0) | 260 (20.8) | <0.001 |
| **CVD** | 709 (56.6) | 760 (60.7) | 741 (59.2) | 698 (55.8) | 0.044 |

Abbreviations: DAC, dietary total antioxidant capacity; HEI-2015, Healthy Eating Index 2015; BMI, body mass index; METs, metabolic equivalent score; CVD, cardiovascular disease. Continuous variables were adjusted for survey weights of NHANES. Categorical variables were unweighted. One-way ANOVA for continuous variables and Chi-square test for categorical variables were performed.

**Supplementary Table 5.** Baseline characteristics of cancer survivors by quartiles of dinner DAC

| **Characteristic** | **Dinner DAC** | | | | ***P* value** |
| --- | --- | --- | --- | --- | --- |
|  | **Q1** | **Q2** | **Q3** | **Q4** |  |
| **Patients, n** | 1253 | 1252 | 1252 | 1252 |  |
| **Age (years)** | 60.95±0.82 | 61.34±0.75 | 61.85±0.69 | 62.39±0.77 | 0.530 |
| **Female** | 527 (42.1) | 745 (59.5) | 679 (54.2) | 671 (53.6) | <0.001 |
| **Race/ethnicity** |  |  |  |  | <0.001 |
| Mexican American | 73 (5.8) | 88 (7.0) | 77 (6.2) | 82 (6.5) |  |
| Non-Hispanic Black | 208 (16.6) | 193 (15.4) | 133 (10.6) | 125 (10.0) |  |
| Non-Hispanic White | 861 (68.7) | 857 (68.5) | 947 (75.6) | 931 (74.4) |  |
| Other Hispanic | 67 (5.3) | 60 (4.8) | 47 (3.8) | 57 (4.6) |  |
| Other | 44 (3.5) | 54 (4.3) | 48 (3.8) | 57 (4.6) |  |
| **Education** |  |  |  |  | <0.001 |
| Less than 9th grade | 125 (10.0) | 142 (11.4) | 100 (8.0) | 97 (7.7) |  |
| 9-11th grade | 166 (13.3) | 174 (13.9) | 151 (12.1) | 111 (8.9) |  |
| College graduate or above | 264 (21.1) | 254 (20.3) | 366 (29.2) | 433 (34.6) |  |
| High school graduate/GED or equivalent | 292 (23.4) | 310 (24.8) | 278 (22.2) | 261 (20.8) |  |
| Some college or Associate of Arts degree | 403 (32.2) | 371 (29.7) | 357 (28.5) | 350 (28.0) |  |
| **Income** |  |  |  |  | <0.001 |
| $ 0 to $ 19,999 | 312 (24.9) | 307 (24.5) | 252 (20.1) | 209 (16.7) |  |
| $20,000 to $44,999 | 388 (31.0) | 403 (32.2) | 371 (29.6) | 351 (28.0) |  |
| $45,000 to $74,999 | 208 (16.6) | 213 (17.0) | 254 (20.3) | 234 (18.7) |  |
| $75,000 to $99,999 | 151 (12.1) | 136 (10.9) | 144 (11.5) | 208 (16.6) |  |
| $100,000 and Over | 194 (15.5) | 193 (15.4) | 231 (18.5) | 250 (20.0) |  |
| **BMI (kg/m^2^)** | 29.06±0.30 | 28.02±0.31 | 28.94±0.30 | 27.91±0.24 | 0.010 |
| **Alcohol intake (g/day)** | 9.40±0.94 | 7.67±0.91 | 5.50±0.56 | 8.22±0.91 | <0.001 |
| **Smoke status** |  |  |  |  | <0.001 |
| Never smoked | 473 (37.7) | 519 (41.5) | 587 (46.9) | 630 (50.3) |  |
| Past smoker | 523 (41.7) | 493 (39.4) | 506 (40.4) | 527 (42.1) |  |
| (continued) |  |  |  |  |  |
| Current smoker | 257 (20.5) | 240 (19.2) | 159 (12.7) | 95 (7.6) |  |
| **Physical activity (METs-h/week)** | 6.86±0.16 | 6.48±0.17 | 6.61±0.17 | 7.62±0.24 | <0.001 |
| **Dietary energy intake (kcal)** | 2148.81±25.21 | 1740.01±46.68 | 1904.47±33.50 | 1980.18±28.92 | <0.001 |
| **Adherence to HEI-2015 score** | 50.02±0.58 | 48.25±0.61 | 52.91±0.57 | 59.82±0.71 | <0.001 |
| **Dietary antioxidant supplement intake (yes)** | 516 (41.2) | 457 (36.5) | 514 (41.1) | 513 (41.0) | 0.042 |
| **Serum HDL-Cholesterol (mg/dL)** | 52.82±0.87 | 57.42±0.86 | 58.00±1.09 | 63.74±1.16 | <0.001 |
| **Serum triglycerides (mmol/L)** | 5.89±0.05 | 5.82±0.05 | 5.90±0.06 | 5.76±0.04 | 0.100 |
| **Glycohemoglobin (%)** | 2.42±0.10 | 2.28±0.11 | 2.32±0.13 | 2.02±0.09 | 0.030 |
| **Hypertension** | 673 (53.7) | 691 (55.2) | 707 (56.5) | 664 (53.0) | 0.311 |
| **Diabetes** | 297 (23.7) | 316 (25.2) | 330 (26.4) | 284 (22.7) | 0.147 |
| **CVD** | 739 (59.0) | 758 (60.5) | 721 (57.6) | 690 (55.1) | 0.042 |

Abbreviations: DAC, dietary total antioxidant capacity; HEI-2015, Healthy Eating Index 2015; BMI, body mass index; METs, metabolic equivalent score; CVD, cardiovascular disease. Continuous variables were adjusted for survey weights of NHANES. Categorical variables were unweighted. One-way ANOVA for continuous variables and Chi-square test for categorical variables were performed.

**Supplementary Table 6.** Baseline characteristics of cancer survivors by quartiles of Δ DAC

| **Characteristic** | Δ **DAC ^a^** | | | | ***P* value** |
| --- | --- | --- | --- | --- | --- |
|  | **Q1** | **Q2** | **Q3** | **Q4** |  |
| **Patients, n** | 1253 | 1252 | 1252 | 1252 |  |
| **Age (years)** | 65.58±0.78 | 62.56±0.64 | 58.92±0.68 | 60.52±0.62 | <0.001 |
| **Female** | 580 (46.3) | 629 (50.2) | 748 (59.7) | 665 (53.1) | <0.001 |
| **Race/ethnicity** |  |  |  |  | <0.001 |
| Mexican American | 64 (5.1) | 83 (6.6) | 85 (6.8) | 88 (7.0) |  |
| Non-Hispanic Black | 154 (12.3) | 211 (16.9) | 155 (12.4) | 139 (11.1) |  |
| Non-Hispanic White | 913 (72.9) | 868 (69.3) | 908 (72.5) | 907 (72.4) |  |
| Other Hispanic | 76 (6.1) | 49 (3.9) | 44 (3.5) | 62 (5.0) |  |
| Other | 46 (3.7) | 41 (3.3) | 60 (4.8) | 56 (4.5) |  |
| **Education** |  |  |  |  | <0.001 |
| Less than 9th grade | 111 (8.9) | 133 (10.6) | 116 (9.3) | 104 (8.3) |  |
| 9-11th grade | 136 (10.9) | 184 (14.7) | 151 (12.1) | 131 (10.5) |  |
| College graduate or above | 362 (28.9) | 283 (22.6) | 303 (24.2) | 369 (29.5) |  |
| High school graduate/GED or equivalent | 280 (22.4) | 287 (23.0) | 308 (24.6) | 266 (21.2) |  |
| Some college or Associate of Arts degree | 363 (29.0) | 363 (29.0) | 373 (29.8) | 382 (30.5) |  |
| **Income** |  |  |  |  | <0.001 |
| $ 0 to $ 19,999 | 274 (21.9) | 314 (25.1) | 262 (20.9) | 230 (18.4) |  |
| $20,000 to $44,999 | 366 (29.2) | 383 (30.6) | 403 (32.2) | 361 (28.8) |  |
| $45,000 to $74,999 | 228 (18.2) | 225 (18.0) | 221 (17.7) | 235 (18.8) |  |
| $75,000 to $99,999 | 182 (14.5) | 137 (10.9) | 142 (11.3) | 178 (14.2) |  |
| $100,000 and Over | 203 (16.2) | 193 (15.4) | 224 (17.9) | 248 (19.8) |  |
| **BMI (kg/m^2^)** | 28.36±0.28 | 28.49±0.26 | 28.54±0.28 | 28.49±0.25 | 0.970 |
| **Alcohol intake (g/day)** | 7.43±0.88 | 7.47±0.72 | 6.75±0.68 | 8.75±1.01 | <0.001 |
| **Smoke status** |  |  |  |  | <0.001 |
| Never smoked | 617 (49.2) | 493 (39.4) | 533 (42.6) | 566 (45.2) |  |
| Past smoker | 530 (42.3) | 528 (42.2) | 456 (36.4) | 535 (42.7) |  |
| (continued) |  |  |  |  |  |
| Current smoker | 106 (8.5) | 231 (18.5) | 263 (21.0) | 151 (12.1) |  |
| **Physical activity (METs-h/week)** | 6.45±0.17 | 6.70±0.16 | 6.74±0.17 | 7.60±0.23 | <0.001 |
| **Dietary energy intake (kcal)** | 2057.75±48.91 | 1893.69±26.32 | 1799.57±32.51 | 2025.37±33.25 | <0.001 |
| **Adherence to HEI-2015 score** | 59.32±0.69 | 50.30±0.52 | 48.38±0.58 | 54.71±0.69 | <0.001 |
| **Dietary antioxidant supplement intake (yes)** | 540 (43.1) | 486 (38.8) | 454 (36.3) | 520 (41.5) | 0.003 |
| **Serum HDL-Cholesterol (mg/dL)** | 60.76±1.01 | 54.14±1.04 | 56.49±0.84 | 61.27±1.26 | <0.001 |
| **Serum triglycerides (mmol/L)** | 5.83±0.04 | 5.92±0.05 | 5.88±0.06 | 5.76±0.04 | 0.06 |
| **Glycohemoglobin (%)** | 2.20±0.09 | 2.37±0.11 | 2.38±0.11 | 2.07±0.10 | 0.11 |
| **Hypertension** | 700 (55.9) | 692 (55.3) | 675 (53.9) | 668 (53.4) | 0.56 |
| **Diabetes** | 283 (22.6) | 320 (25.6) | 318 (25.4) | 306 (24.4) | 0.285 |
| **CVD** | 765 (61.1) | 749 (59.8) | 708 (56.5) | 686 (54.8) | 0.005 |

Abbreviations: DAC, dietary total antioxidant capacity; HEI-2015, Healthy Eating Index 2015; BMI, body mass index; METs, metabolic equivalent score; CVD, cardiovascular disease. Continuous variables were adjusted for survey weights of NHANES. Categorical variables were unweighted. a Δ equals dinner DAC minus breakfast DAC. One-way ANOVA for continuous variables and Chi-square test for categorical variables were performed.

**Supplementary Table 7.** Associations of all-cause mortality with quartiles of DAC distribution across a day in cancer survivors

| **Exposure** | **Case/N** | **Model 1** | | **Model 2** | | **Model 3** | |
| --- | --- | --- | --- | --- | --- | --- | --- |
|  |  | **HR (95% CI)** | ***P* value** | **HR (95% CI)** | ***P* value** | **HR (95% CI)** | ***P* value** |
| **Total DAC** |  |  |  |  |  |  |  |
| Continuous total DAC | 1811/5009 | 0.93 (0.90, 0.96) | <0.001 | 0.96 (0.93, 0.99) | 0.006 | 0.99 (0.95, 1.02) | 0.515 |
| Q1 (<2.19) | 396/1253 | 1.00 (Reference) |  | 1.00 (Reference) |  | 1.00 (Reference) |  |
| Q2 (2.19 to 3.47) | 468/1252 | 0.84 (0.68, 1.03) |  | 0.87 (0.69, 1.10) |  | 0.93 (0.73, 1.17) |  |
| Q3 (3.47 to 5.50) | 453/1252 | 0.65 (0.52, 0.81) |  | 0.73 (0.58, 0.93) |  | 0.82 (0.64, 1.05) |  |
| Q4 (>5.50) | 494/1252 | 0.57 (0.47, 0.71) | <0.001* | 0.71 (0.58, 0.88) | <0.001* | 0.86 (0.67, 1.10) | 0.143* |
| **Breakfast DAC (mmol)** |  |  |  |  |  |  |  |
| Continuous breakfast DAC | 1811/5009 | 0.93 (0.88, 0.97) | 0.001 | 0.96 (0.92, 1.01) | 0.083 | 1.01 (0.96, 1.06) | 0.815 |
| Q1 (<0.27) | 360/1253 | 1.00 (Reference) |  | 1.00 (Reference) |  | 1.00 (Reference) |  |
| Q2 (0.27 to 0.62) | 423/1252 | 0.87 (0.71, 1.06) |  | 0.86 (0.69, 1.06) |  | 0.89 (0.71, 1.10) |  |
| Q3 (0.62 to 1.74) | 494/1252 | 0.90 (0.75, 1.09) |  | 1.01 (0.84, 1.22) |  | 1.11 (0.92, 1.35) |  |
| Q4 (>1.74) | 534/1252 | 0.72 (0.60, 0.87) | 0.002* | 0.84 (0.69, 1.01) | 0.214* | 1.01 (0.81, 1.27) | 0.465* |
| **Lunch DAC (mmol)** |  |  |  |  |  |  |  |
| Continuous lunch DAC | 1811/5009 | 0.93 (0.89, 0.97) | 0.002 | 0.96 (0.92, 1.01) | 0.109 | 0.98 (0.93, 1.03) | 0.420 |
| Q1 (<0.72) | 411/1253 | 1.00 (Reference) |  | 1.00 (Reference) |  | 1.00 (Reference) |  |
| Q2 (0.72 to 1.38) | 473/1252 | 0.99 (0.82, 1.19) |  | 0.94 (0.78, 1.15) |  | 0.97 (0.79, 1.18) |  |
| Q3 (1.38 to 2.47) | 474/1252 | 0.82 (0.67, 1.01) |  | 0.88 (0.71, 1.08) |  | 0.92 (0.73, 1.15) |  |
| Q4 (>2.47) | 453/1252 | 0.76 (0.63, 0.92) | 0.001* | 0.86 (0.70, 1.05) | 0.113* | 0.93 (0.74, 1.17) | 0.452* |
| **Dinner DAC (mmol)** |  |  |  |  |  |  |  |
| Continuous dinner DAC | 1811/5009 | 0.91 (0.85, 0.98) | 0.016 | 0.96 (0.89, 1.02) | 0.203 | 0.98 (0.91, 1.06) | 0.580 |
| Q1 (<0.25) | 440/1253 | 1.00 (Reference) |  | 1.00 (Reference) |  | 1.00 (Reference) |  |
| Q2 (0.25 to 0.70) | 469/1252 | 0.95 (0.78, 1.15) |  | 0.92 (0.76, 1.12) |  | 0.93 (0.76, 1.14) |  |
| Q3 (0.70 to 1.54) | 452/1252 | 0.81 (0.65, 1.00) |  | 0.84 (0.68, 1.03) |  | 0.83 (0.67, 1.02) |  |
| Q4 (>1.54) | 450/1252 | 0.66 (0.54, 0.81) | <0.001* | 0.74 (0.61, 0.91) | 0.002* | 0.79 (0.65, 0.98) | 0.017* |
| **Δ DAC ^a^** |  |  |  |  |  |  |  |
| (continued) |  |  |  |  |  |  |  |
| Continuous Δ DAC | 1811/5009 | 0.93 (0.87, 1.00) | 0.063 | 0.95 (0.88, 1.03) | 0.221 | 0.96 (0.88, 1.04) | 0.289 |
| Q1 (<-0.84) | 513/1253 | 1.00 (Reference) |  | 1.00 (Reference) |  | 1.00 (Reference) |  |
| Q2 (-0.84 to -0.01) | 488/1252 | 0.84 (0.61, 1.18) |  | 0.83 (0.59, 1.17) |  | 0.82 (0.58, 1.16) |  |
| Q3 (-0.01 to 0.73) | 399/1252 | 0.80 (0.58, 1.10) |  | 0.85 (0.61, 1.18) |  | 0.86 (0.62, 1.18) |  |
| Q4 (>0.73) | 411/1252 | 0.71 (0.53, 0.95) | 0.017* | 0.77 (0.58, 1.03) | 0.107* | 0.77 (0.56, 1.06) | 0.138* |

Abbreviations: HR, hazard ratio; CI, confidence intervals; DAC, dietary total antioxidant capacity; HEI-2015, Healthy Eating Index 2015; BMI, body mass index; METs, metabolic equivalent score; CVD, cardiovascular disease. * *P* for trend across the quartile of DAC. HR with 95%CI was assessed by weighted Cox regression analyses. Model 1 adjusted for, age, sex, and race. Model 2 further adjusted for education, family income, dietary energy intake, alcohol consumption per day, smoke status, METs, and BMI. Model 3 further adjusted for serum HDL-Cholesterol, serum triglycerides, serum glycohemoglobin, diabetes, hypertension, CVD, dietary antioxidant supplement intake (vitamin C or vitamin E), and adherence to HEI-2015 score. Models for DAC from breakfast, lunch, and dinner were further adjusted except the one that defined the group. a Δ equals dinner DAC minus breakfast DAC.

**Supplementary Table 8.** Associations of cancer mortality with quartiles of DAC distribution across a day in cancer survivors

| **Exposure** | **Case/N** | **Model 1** | | **Model 2** | | **Model 3** | |
| --- | --- | --- | --- | --- | --- | --- | --- |
|  |  | **HR (95% CI)** | ***P* value** | **HR (95% CI)** | ***P* value** | **HR (95% CI)** | ***P* value** |
| **Total DAC** |  |  |  |  |  |  |  |
| Continuous total DAC | 575/5009 | 0.95 (0.88, 1.01) | 0.107 | 0.98 (0.92, 1.04) | 0.508 | 1.01 (0.94, 1.08) | 0.792 |
| Q1 (<2.19) | 150/1253 | 1.00 (Reference) |  | 1.00 (Reference) |  | 1.00 (Reference) |  |
| Q2 (2.19 to 3.47) | 149/1252 | 0.79 (0.53, 1.17) |  | 0.88 (0.58, 1.33) |  | 0.93 (0.61, 1.43) |  |
| Q3 (3.47 to 5.50) | 141/1252 | 0.68 (0.46, 1.00) |  | 0.79 (0.53, 1.19) |  | 0.88 (0.58, 1.33) |  |
| Q4 (>5.50) | 135/1252 | 0.58 (0.39, 0.85) | 0.004* | 0.73 (0.49, 1.10) | 0.107* | 0.89 (0.58, 1.37) | 0.548* |
| **Breakfast DAC (mmol)** |  |  |  |  |  |  |  |
| Continuous breakfast DAC | 575/5009 | 0.93 (0.84, 1.03) | 0.183 | 0.97 (0.87, 1.07) | 0.521 | 1.02 (0.92, 1.12) | 0.774 |
| Q1 (<0.27) | 134/1253 | 1.00 (Reference) |  | 1.00 (Reference) |  | 1.00 (Reference) |  |
| Q2 (0.27 to 0.62) | 146/1252 | 0.89 (0.61, 1.30) |  | 0.93 (0.63, 1.40) |  | 0.91 (0.62, 1.34) |  |
| Q3 (0.62 to 1.74) | 145/1252 | 0.87 (0.60, 1.24) |  | 1.01 (0.69, 1.46) |  | 1.11 (0.77, 1.61) |  |
| Q4 (>1.74) | 150/1252 | 0.70 (0.51, 0.96) | 0.024* | 0.82 (0.58, 1.16) | 0.321* | 0.97 (0.67, 1.41) | 0.874* |
| **Lunch DAC (mmol)** |  |  |  |  |  |  |  |
| Continuous lunch DAC | 575/5009 | 0.93 (0.85, 1.01) | 0.094 | 0.96 (0.88, 1.04) | 0.31 | 0.98 (0.90, 1.07) | 0.659 |
| Q1 (<0.72) | 147/1253 | 1.00 (Reference) |  | 1.00 (Reference) |  | 1.00 (Reference) |  |
| Q2 (0.72 to 1.38) | 156/1252 | 1.00 (0.70, 1.43) |  | 0.96 (0.67, 1.36) |  | 1.02 (0.71, 1.49) |  |
| Q3 (1.38 to 2.47) | 136/1252 | 0.76 (0.52, 1.11) |  | 0.81 (0.56, 1.18) |  | 0.89 (0.60, 1.32) |  |
| Q4 (>2.47) | 136/1252 | 0.82 (0.54, 1.25) | 0.210* | 0.91 (0.60, 1.38) | 0.534* | 1.00 (0.64, 1.57) | 0.862* |
| **Dinner DAC (mmol)** |  |  |  |  |  |  |  |
| Continuous dinner DAC | 575/5009 | 0.98 (0.84, 1.13) | 0.75 | 1.02 (0.90, 1.16) | 0.756 | 1.05 (0.92, 1.20) | 0.471 |
| Q1 (<0.25) | 151/1253 | 1.00 (Reference) |  | 1.00 (Reference) |  | 1.00 (Reference) |  |
| Q2 (0.25 to 0.70) | 145/1252 | 0.94 (0.64, 1.38) |  | 0.93 (0.63, 1.37) |  | 0.92 (0.61, 1.38) |  |
| Q3 (0.70 to 1.54) | 127/1252 | 0.66 (0.42, 1.04) |  | 0.72 (0.46, 1.13) |  | 0.73 (0.46, 1.15) |  |
| Q4 (>1.54) | 152/1252 | 0.74 (0.51, 1.07) | 0.047* | 0.86 (0.60, 1.23) | 0.272* | 0.94 (0.65, 1.36) | 0.530* |
| **Δ DAC ^a^** |  |  |  |  |  |  |  |
| (continued) |  |  |  |  |  |  |  |
| Continuous Δ DAC | 575/5009 | 1.10 (0.94, 1.28) | 0.223 | 1.11 (0.96, 1.28) | 0.161 | 1.13 (0.97, 1.30) | 0.114 |
| Q1 (<-0.84) | 148/1253 | 1.00 (Reference) |  | 1.00 (Reference) |  | 1.00 (Reference) |  |
| Q2 (-0.84 to -0.01) | 157/1252 | 1.40 (0.83, 2.36) |  | 1.32 (0.77, 2.28) |  | 1.31 (0.75, 2.30) |  |
| Q3 (-0.01 to 0.73) | 130/1252 | 0.81 (0.48, 1.38) |  | 0.82 (0.48, 1.39) |  | 0.82 (0.46, 1.47) |  |
| Q4 (>0.73) | 140/1252 | 1.29 (0.76, 2.16) | 0.833* | 1.30 (0.77, 2.19) | 0.729* | 1.37 (0.78, 2.43) | 0.606* |

Abbreviations: HR, hazard ratio; CI, confidence intervals; DAC, dietary total antioxidant capacity; HEI-2015, Healthy Eating Index 2015; BMI, body mass index; METs, metabolic equivalent score; CVD, cardiovascular disease. * *P* for trend across the quartile of DAC. HR with 95%CI was assessed by weighted Cox regression analyses. Model 1 adjusted for, age, sex, and race. Model 2 further adjusted for education, family income, dietary energy intake, alcohol consumption per day, smoke status, METs, and BMI. Model 3 further adjusted for serum HDL-Cholesterol, serum triglycerides, serum glycohemoglobin, diabetes, hypertension, CVD, dietary antioxidant supplement intake (vitamin C or vitamin E), and adherence to HEI-2015 score. Models for DAC from breakfast, lunch, and dinner were further adjusted except the one that defined the group. a Δ equals dinner DAC minus breakfast DAC.

**Supplementary Table 9.** Associations of noncancer mortality with quartiles of DAC distribution across a day in cancer survivors

| **Exposure** | **Case/N** | **Model 1** | | **Model 2** | | **Model 3** | |
| --- | --- | --- | --- | --- | --- | --- | --- |
|  |  | **HR (95% CI)** | ***P* value** | **HR (95% CI)** | ***P* value** | **HR (95% CI)** | ***P* value** |
| **Total DAC** |  |  |  |  |  |  |  |
| Continuous total DAC | 1236/5009 | 0.92 (0.89, 0.95) | <0.001 | 0.95 (0.92, 0.98) | 0.003 | 0.97 (0.94, 1.01) | 0.203 |
| Q1 (<2.19) | 246/1253 | 1.00 (Reference) |  | 1.00 (Reference) |  | 1.00 (Reference) |  |
| Q2 (2.19 to 3.47) | 319/1252 | 0.86 (0.69, 1.07) |  | 0.87 (0.69, 1.10) |  | 0.90 (0.71, 1.14) |  |
| Q3 (3.47 to 5.50) | 312/1252 | 0.64 (0.50, 0.82) |  | 0.71 (0.54, 0.92) |  | 0.82 (0.64, 1.05) |  |
| Q4 (>5.50) | 359/1252 | 0.57 (0.46, 0.71) | <0.001* | 0.70 (0.57, 0.87) | <0.001* | 0.76 (0.60, 0.92) | 0.009* |
| **Breakfast DAC (mmol)** |  |  |  |  |  |  |  |
| Continuous breakfast DAC | 1236/5009 | 0.92 (0.87, 0.98) | 0.005 | 0.96 (0.91, 1.01) | 0.117 | 1.00 (0.94, 1.06) | 0.957 |
| Q1 (<0.27) | 226/1253 | 1.00 (Reference) |  | 1.00 (Reference) |  | 1.00 (Reference) |  |
| Q2 (0.27 to 0.62) | 277/1252 | 0.86 (0.67, 1.09) |  | 0.82 (0.64, 1.06) |  | 0.88 (0.68, 1.13) |  |
| Q3 (0.62 to 1.74) | 349/1252 | 0.93 (0.75, 1.15) |  | 1.02 (0.83, 1.26) |  | 1.11 (0.89, 1.38) |  |
| Q4 (>1.74) | 384/1252 | 0.74 (0.57, 0.94) | 0.027* | 0.84 (0.66, 1.07) | 0.420* | 1.02 (0.77, 1.36) | 0.501* |
| **Lunch DAC (mmol)** |  |  |  |  |  |  |  |
| Continuous lunch DAC | 1236/5009 | 0.93 (0.89, 0.98) | 0.011 | 0.97 (0.92, 1.02) | 0.26 | 0.98 (0.92, 1.04) | 0.477 |
| Q1 (<0.72) | 264/1253 | 1.00 (Reference) |  | 1.00 (Reference) |  | 1.00 (Reference) |  |
| Q2 (0.72 to 1.38) | 317/1252 | 0.98 (0.78, 1.24) |  | 0.94 (0.74, 1.19) |  | 0.94 (0.74, 1.19) |  |
| Q3 (1.38 to 2.47) | 338/1252 | 0.85 (0.69, 1.06) |  | 0.91 (0.72, 1.15) |  | 0.92 (0.73, 1.17) |  |
| Q4 (>2.47) | 317/1252 | 0.74 (0.59, 0.92) | 0.002* | 0.84 (0.66, 1.08) | 0.168* | 0.89 (0.68, 1.15) | 0.366* |
| **Dinner DAC (mmol)** |  |  |  |  |  |  |  |
| Continuous dinner DAC | 1236/5009 | 0.88 (0.82, 0.94) | <0.001 | 0.92 (0.86, 0.98) | 0.011 | 0.93 (0.87, 1.00) | 0.058 |
| Q1 (<0.25) | 289/1253 | 1.00 (Reference) |  | 1.00 (Reference) |  | 1.00 (Reference) |  |
| Q2 (0.25 to 0.70) | 324/1252 | 0.95 (0.75, 1.21) |  | 0.91 (0.71, 1.17) |  | 0.92 (0.71, 1.19) |  |
| Q3 (0.70 to 1.54) | 325/1252 | 0.88 (0.71, 1.09) |  | 0.89 (0.72, 1.10) |  | 0.86 (0.69, 1.08) |  |
| Q4 (>1.54) | 298/1252 | 0.62 (0.50, 0.77) | <0.001* | 0.69 (0.55, 0.85) | <0.001* | 0.72 (0.57, 0.90) | 0.003* |
| **Δ DAC ^a^** |  |  |  |  |  |  |  |
| Continuous Δ DAC | 1236/5009 | 0.87 (0.81, 0.95) | 0.001 | 0.89 (0.82, 0.98) | 0.013 | 0.89 (0.81, 0.98) | 0.016 |
| (continued) |  |  |  |  |  |  |  |
| Q1 (<-0.84) | 365/1253 | 1.00 (Reference) |  | 1.00 (Reference) |  | 1.00 (Reference) |  |
| Q2 (-0.84 to -0.01) | 331/1252 | 0.68 (0.46, 0.99) |  | 0.69 (0.46, 1.02) |  | 0.70 (0.47, 1.03) |  |
| Q3 (-0.01 to 0.73) | 269/1252 | 0.79 (0.53, 1.16) |  | 0.87 (0.59, 1.29) |  | 0.90 (0.62, 1.32) |  |
| Q4 (>0.73) | 271/1252 | 0.53 (0.37, 0.76) | 0.004* | 0.59 (0.41, 0.84) | 0.029* | 0.56 (0.38, 0.83) | 0.022* |

Abbreviations: HR, hazard ratio; CI, confidence intervals; DAC, dietary total antioxidant capacity; HEI-2015, Healthy Eating Index 2015; BMI, body mass index; METs, metabolic equivalent score; CVD, cardiovascular disease. * *P* for trend across the quartile of DAC. HR with 95%CI was assessed by weighted Cox regression analyses. Model 1 adjusted for, age, sex, and race. Model 2 further adjusted for education, family income, dietary energy intake, alcohol consumption per day, smoke status, METs, and BMI. Model 3 further adjusted for serum HDL-Cholesterol, serum triglycerides, serum glycohemoglobin, diabetes, hypertension, CVD, dietary antioxidant supplement intake (vitamin C or vitamin E), and adherence to HEI-2015 score. Models for DAC from breakfast, lunch, and dinner were further adjusted except the one that defined the group. a Δ equals dinner DAC minus breakfast DAC.

**Supplementary Table 10.** HR with 95% CI of nine non-cancer mortality according to quartiles of dinner DAC in cancer survivors

| **Cause of non-cancer mortality** | **Case/N** | **Model 1** | | **Model 2** | | **Model 3** | |
| --- | --- | --- | --- | --- | --- | --- | --- |
|  |  | **HR (95% CI)** | ***P* value** | **HR (95% CI)** | ***P* value** | **HR (95% CI)** | ***P* value** |
| **Chronic lower respiratory diseases** |  |  |  |  |  |  |  |
| Continuous dinner DAC | 101/5009 | 0.82 (0.64, 1.04) | 0.098 | 0.92 (0.74, 1.14) | 0.441 | 0.94 (0.76, 1.17) | 0.605 |
| Q1 (<0.25) | 28/1253 | 1.00 (Reference) |  | 1.00 (Reference) |  | 1.00 (Reference) |  |
| Q2 (0.25 to 0.70) | 29/1252 | 0.85 (0.43, 1.68) |  | 1.07 (0.49, 2.33) |  | 0.99 (0.46, 2.15) |  |
| Q3 (0.70 to 1.54) | 22/1252 | 0.70 (0.29, 1.68) |  | 0.89 (0.33, 2.44) |  | 0.87 (0.31, 2.45) |  |
| Q4 (>1.54) | 22/1252 | 0.53 (0.24, 1.19) | 0.127* | 0.82 (0.35, 1.95) | 0.606* | 0.88 (0.37, 2.08) | 0.734* |
|  |  |  |  |  |  |  |  |
| **Nephritis, nephrotic syndrome and nephrosis** |  |  |  |  |  |  |  |
| Continuous dinner DAC | 31/5009 | 0.79 (0.54, 1.13) | 0.197 | 0.79 (0.54, 1.15) | 0.221 | 0.82 (0.57, 1.17) | 0.270 |
| Q1 (<0.25) | 9/1253 | 1.00 (Reference) |  | 1.00 (Reference) |  | 1.00 (Reference) |  |
| Q2 (0.25 to 0.70) | 6/1252 | 0.12 (0.03, 0.50) |  | 0.08 (0.02, 0.34) |  | 0.17 (0.05, 0.65) |  |
| Q3 (0.70 to 1.54) | 8/1252 | 0.75 (0.23, 2.40) |  | 0.58 (0.19, 1.70) |  | 0.54 (0.15, 1.93) |  |
| Q4 (>1.54) | 8/1252 | 0.42 (0.13, 1.36) | 0.555* | 0.37 (0.14, 1.01) | 0.489* | 0.57 (0.20, 1.64) | 0.688* |
|  |  |  |  |  |  |  |  |
| **Influenza and pneumonia** |  |  |  |  |  |  |  |
| Continuous dinner DAC | 30/5009 | 0.57 (0.34, 0.97) | 0.038 | 0.63 (0.36, 1.11) | 0.108 | 0.63 (0.38, 1.04) | 0.071 |
| Q1 (<0.25) | 8/1253 | 1.00 (Reference) |  | 1.00 (Reference) |  | 1.00 (Reference) |  |
| Q2 (0.25 to 0.70) | 12/1252 | 1.32 (0.40, 4.29) |  | 1.15 (0.34, 3.91) |  | 1.05 (0.33, 3.33) |  |
| Q3 (0.70 to 1.54) | 7/1252 | 0.58 (0.16, 2.01) |  | 0.57 (0.16, 2.04) |  | 0.51 (0.16, 1.62) |  |
| Q4 (>1.54) | 3/1252 | 0.31 (0.07, 1.42) | 0.028* | 0.40 (0.09, 1.85) | 0.096* | 0.39 (0.10, 1.46) | 0.049* |
|  |  |  |  |  |  |  |  |
| **Accidents (unintentional injuries)** |  |  |  |  |  |  |  |
| (continued) |  |  |  |  |  |  |  |
| Continuous dinner DAC | 49/5009 | 0.68 (0.42, 1.11) | 0.123 | 0.75 (0.46, 1.24) | 0.267 | 0.74 (0.44, 1.25) | 0.265 |
| Q1 (<0.25) | 10/1253 | 1.00 (Reference) |  | 1.00 (Reference) |  | 1.00 (Reference) |  |
| Q2 (0.25 to 0.70) | 20/1252 | 0.81 (0.23, 2.83) |  | 0.90 (0.30, 2.73) |  | 0.79 (0.31, 2.02) |  |
| Q3 (0.70 to 1.54) | 6/1252 | 0.14 (0.03, 0.56) |  | 0.14 (0.03, 0.62) |  | 0.12 (0.03, 0.49) |  |
| Q4 (>1.54) | 13/1252 | 0.39 (0.13, 1.14) | 0.035* | 0.52 (0.19, 1.43) | 0.076* | 0.47 (0.18, 1.25) | 0.044* |
|  |  |  |  |  |  |  |  |
| **Diabetes mellitus** |  |  |  |  |  |  |  |
| Continuous dinner DAC | 47/5009 | 0.93 (0.70, 1.25) | 0.646 | 1.00 (0.71, 1.41) | 0.993 | 1.09 (0.69, 1.71) | 0.709 |
| Q1 (<0.25) | 8/1253 | 1.00 (Reference) |  | 1.00 (Reference) |  | 1.00 (Reference) |  |
| Q2 (0.25 to 0.70) | 10/1252 | 0.51 (0.11, 2.40) |  | 0.48 (0.13, 1.82) |  | 0.62 (0.22, 1.74) |  |
| Q3 (0.70 to 1.54) | 14/1252 | 1.61 (0.48, 5.42) |  | 1.42 (0.51, 3.94) |  | 1.13 (0.37, 3.45) |  |
| Q4 (>1.54) | 15/1252 | 0.72 (0.18, 2.84) | 0.933* | 0.82 (0.24, 2.77) | 0.709* | 1.19 (0.34, 4.17) | 0.534* |
|  |  |  |  |  |  |  |  |
| **Diseases of heart** |  |  |  |  |  |  |  |
| Continuous dinner DAC | 400/5009 | 0.85 (0.76, 0.95) | 0.004 | 0.88 (0.79, 0.99) | 0.031 | 0.86 (0.76, 0.98) | 0.019 |
| Q1 (<0.25) | 86/1253 | 1.00 (Reference) |  | 1.00 (Reference) |  | 1.00 (Reference) |  |
| Q2 (0.25 to 0.70) | 112/1252 | 1.37 (0.90, 2.10) |  | 1.30 (0.83, 2.01) |  | 1.37 (0.88, 2.12) |  |
| Q3 (0.70 to 1.54) | 104/1252 | 1.06 (0.73, 1.54) |  | 1.02 (0.71, 1.46) |  | 1.01 (0.68, 1.50) |  |
| Q4 (>1.54) | 98/1252 | 0.77 (0.49, 1.21) | 0.061* | 0.84 (0.53, 1.32) | 0.157* | 0.82 (0.52, 1.29) | 0.131* |
|  |  |  |  |  |  |  |  |
| **Cerebrovascular diseases** |  |  |  |  |  |  |  |
| Continuous dinner DAC | 93/5009 | 1.03 (0.82, 1.31) | 0.773 | 1.03 (0.82, 1.28) | 0.82 | 1.08 (0.84, 1.38) | 0.554 |
| Q1 (<0.25) | 18/1253 | 1.00 (Reference) |  | 1.00 (Reference) |  | 1.00 (Reference) |  |
| Q2 (0.25 to 0.70) | 21/1252 | 0.72 (0.30, 1.73) |  | 0.77 (0.39, 1.52) |  | 0.85 (0.41, 1.76) |  |
| (continued) |  |  |  |  |  |  |  |
| Q3 (0.70 to 1.54) | 25/1252 | 1.31 (0.48, 3.55) |  | 1.38 (0.55, 3.45) |  | 1.61 (0.72, 3.62) |  |
| Q4 (>1.54) | 29/1252 | 0.73 (0.29, 1.84) | 0.784* | 0.77 (0.33, 1.76) | 0.846* | 0.92 (0.36, 2.31) | 0.821* |
|  |  |  |  |  |  |  |  |
| **Alzheimer's disease** |  |  |  |  |  |  |  |
| Continuous dinner DAC | 66/5009 | 0.82 (0.59, 1.15) | 0.245 | 0.85 (0.61, 1.18) | 0.335 | 0.92 (0.67, 1.28) | 0.635 |
| Q1 (<0.25) | 25/1253 | 1.00 (Reference) |  | 1.00 (Reference) |  | 1.00 (Reference) |  |
| Q2 (0.25 to 0.70) | 12/1252 | 0.40 (0.18, 0.92) |  | 0.35 (0.14, 0.87) |  | 0.34 (0.14, 0.84) |  |
| Q3 (0.70 to 1.54) | 15/1252 | 0.32 (0.14, 0.73) |  | 0.29 (0.12, 0.70) |  | 0.29 (0.13, 0.67) |  |
| Q4 (>1.54) | 14/1252 | 0.30 (0.13, 0.70) | 0.010* | 0.31 (0.13, 0.76) | 0.019* | 0.38 (0.16, 0.88) | 0.028* |
|  |  |  |  |  |  |  |  |
| **All other causes** |  |  |  |  |  |  |  |
| Continuous dinner DAC | 423/5009 | 0.90 (0.80, 1.01) | 0.064 | 0.93 (0.82, 1.04) | 0.208 | 0.95 (0.83, 1.08) | 0.446 |
| Q1 (<0.25) | 101/1253 | 1.00 (Reference) |  | 1.00 (Reference) |  | 1.00 (Reference) |  |
| Q2 (0.25 to 0.70) | 102/1252 | 0.83 (0.56, 1.22) |  | 0.75 (0.50, 1.13) |  | 0.75 (0.50, 1.13) |  |
| Q3 (0.70 to 1.54) | 123/1252 | 0.82 (0.58, 1.16) |  | 0.81 (0.57, 1.15) |  | 0.83 (0.57, 1.20) |  |
| Q4 (>1.54) | 97/1252 | 0.55 (0.40, 0.76) | <0.001* | 0.59 (0.42, 0.82) | 0.004* | 0.62 (0.43, 0.90) | 0.025* |

Abbreviations: HR, hazard ratio; CI, confidence intervals; DAC, dietary total antioxidant capacity; HEI-2015, Healthy Eating Index 2015; BMI, body mass index; METs, metabolic equivalent score; CVD, cardiovascular disease. * *P* for trend across the quartile of DAC. HR with 95%CI was assessed by weighted Cox regression analyses. Model 1 adjusted for, age, sex, and race. Model 2 further adjusted for education, family income, dietary energy intake, alcohol consumption per day, smoke status, METs, and BMI. Model 3 further adjusted for serum HDL-Cholesterol, serum triglycerides, serum glycohemoglobin, diabetes, hypertension, CVD, dietary antioxidant supplement intake (vitamin C or vitamin E), adherence to HEI-2015 score, breakfast DAC and lunch DAC.

**Supplementary Table 11.** HR with 95% CI of non-cancer mortality according to quartiles of dinner DAC stratified to age, sex, and BMI

| **Biomarker** | **Case/N** | **Dinner DAC** | | | | ***P* for trend*** |
| --- | --- | --- | --- | --- | --- | --- |
|  |  | **Q1** | **Q2** | **Q3** | **Q4** |  |
| **Age, years** |  |  |  |  |  |  |
| <60 | 157/1442 | 1 | 0.80 (0.28, 2.24) | 1.01 (0.38, 2.69) | 0.45 (0.16, 1.24) | 0.279 |
| ≥60 | 1652/3567 | 1 | 1.02 (0.78, 1.33) | 0.95 (0.74, 1.22) | 0.90 (0.71, 1.14) | 0.271 |
| ***P* for interaction** |  |  |  |  |  | 0.077 |
| **Sex** |  |  |  |  |  |  |
| Male | 1060/2387 | 1 | 0.97 (0.68, 1.36) | 0.98 (0.73, 1.31) | 0.77 (0.57, 1.03) | 0.116 |
| Female | 749/2622 | 1 | 0.89 (0.60, 1.32) | 0.78 (0.53, 1.14) | 0.68 (0.48, 0.97) | 0.017 |
| ***P* for interaction** |  |  |  |  |  | 0.657 |
| **BMI, kg/m**2 |  |  |  |  |  |  |
| <25.0 | 671/1517 | 1 | 0.84 (0.57, 1.25) | 1.05 (0.7, 1.56) | 0.71 (0.45, 1.11) | 0.265 |
| 25.0-30.0 | 666/1828 | 1 | 1.09 (0.71, 1.67) | 1.12 (0.79, 1.59) | 0.82 (0.54, 1.23) | 0.343 |
| >30.0 | 472/1664 | 1 | 0.94 (0.57, 1.57) | 0.61 (0.38, 1) | 0.68 (0.43, 1.08) | 0.040 |
| ***P* for interaction** |  |  |  |  |  | 0.552 |

Abbreviations: HR, hazard ratio; CI, confidence intervals; DAC, dietary total antioxidant capacity; HEI-2015, Healthy Eating Index 2015; BMI, body mass index; METs, metabolic equivalent score; CVD, cardiovascular disease. * *P* for trend across the quartile of DAC. HR with 95%CI was assessed by weighted Cox regression analyses. Model was adjusted for race, education, smoking status, alcohol consumption per day, family income, dietary energy intake, METs, serum HDL-Cholesterol, serum triglycerides, serum glycohemoglobin, diabetes, hypertension, CVD, dietary antioxidant supplement intake (vitamin C or vitamin E), adherence to HEI-2015 score, breakfast DAC and lunch DAC. Models for subgroup were further adjusted except the one that defined the group.

**Supplementary Table 12.** HR with 95% CI of all-cause, cancer, and non-cancer mortality by quartiles of total DAC after excluding dinner

| **Outcome** | **Case/N** | **Model 1** | | **Model 2** | | **Model 3** | |
| --- | --- | --- | --- | --- | --- | --- | --- |
|  |  | **HR (95% CI)** | ***P* value** | **HR (95% CI)** | ***P* value** | **HR (95% CI)** | ***P* value** |
| **All-cause mortality** |  |  |  |  |  |  |  |
| Continuous total DAC | 1811/5009 | 0.93 (0.90, 0.96) | <0.001 | 0.96 (0.94, 0.99) | 0.011 | 0.99 (0.96, 1.03) | 0.666 |
| Q1 (<1.38) | 384/1253 | 1.00 (Reference) |  | 1.00 (Reference) |  | 1.00 (Reference) |  |
| Q2 (1.38 to 2.45) | 458/1252 | 0.94 (0.77, 1.14) |  | 1.04 (0.85, 1.27) |  | 1.09 (0.89, 1.33) |  |
| Q3 (2.45 to 4.10) | 469/1252 | 0.76 (0.62, 0.94) |  | 0.87 (0.69, 1.10) |  | 0.95 (0.76, 1.20) |  |
| Q4 (>4.10) | 500/1252 | 0.65 (0.55, 0.78) | <0.001* | 0.82 (0.69, 0.98) | 0.007* | 0.96 (0.79, 1.18) | 0.455* |
| **Cancer mortality** |  |  |  |  |  |  |  |
| Continuous total DAC | 575/5009 | 0.93 (0.88, 0.99) | 0.032 | 0.96 (0.90, 1.03) | 0.236 | 0.99 (0.93, 1.05) | 0.743 |
| Q1 (<1.38) | 139/1253 | 1.00 (Reference) |  | 1.00 (Reference) |  | 1.00 (Reference) |  |
| Q2 (1.38 to 2.45) | 155/1252 | 0.94 (0.68, 1.30) |  | 1.08 (0.77, 1.50) |  | 1.14 (0.81, 1.59) |  |
| Q3 (2.45 to 4.10) | 148/1252 | 0.91 (0.63, 1.32) |  | 1.05 (0.71, 1.56) |  | 1.16 (0.78, 1.72) |  |
| Q4 (>4.10) | 133/1252 | 0.64 (0.44, 0.94) | 0.022* | 0.80 (0.54, 1.20) | 0.275* | 0.94 (0.62, 1.42) | 0.830* |
| **Non-cancer mortality** |  |  |  |  |  |  |  |
| Continuous total DAC | 1236/5009 | 0.93 (0.90, 0.96) | <0.001 | 0.96 (0.93, 1.00) | 0.037 | 0.99 (0.95, 1.03) | 0.706 |
| Q1 (<1.38) | 245/1253 | 1.00 (Reference) |  | 1.00 (Reference) |  | 1.00 (Reference) |  |
| Q2 (1.38 to 2.45) | 303/1252 | 0.94 (0.74, 1.18) |  | 1.02 (0.80, 1.30) |  | 1.06 (0.83, 1.34) |  |
| Q3 (2.45 to 4.10) | 321/1252 | 0.70 (0.56, 0.87) |  | 0.80 (0.63, 1.01) |  | 0.86 (0.69, 1.08) |  |
| Q4 (>4.10) | 367/1252 | 0.65 (0.53, 0.80) | <0.001* | 0.82 (0.67, 1.01) | 0.010* | 0.95 (0.76, 1.19) | 0.320* |

Abbreviations: HR, hazard ratio; CI, confidence intervals; DAC, dietary total antioxidant capacity; HEI-2015, Healthy Eating Index 2015; BMI, body mass index; METs, metabolic equivalent score; CVD, cardiovascular disease. * *P* for trend across the quartile of DAC. HR with 95%CI was assessed by weighted Cox regression analyses. Model 1 adjusted for, age, sex, and race. Model 2 further adjusted for education, family income, dietary energy intake, alcohol consumption per day, smoke status, METs, and BMI. Model 3 further adjusted for serum HDL-Cholesterol, serum triglycerides, serum glycohemoglobin, diabetes, hypertension, CVD, dietary antioxidant supplement intake (vitamin C or vitamin E), and adherence to HEI-2015 score. Models for DAC from breakfast, lunch, and dinner were further adjusted except the one that defined the group.

**Supplementary Table 13.** Risk for all-cause, cancer and noncancer mortality by quartiles of DAC after including snack among cancer survivors

| **Exposure** | **All-cause mortality** | | **Cancer mortality** | | **Noncancer mortality** | |
| --- | --- | --- | --- | --- | --- | --- |
|  | **HR (95% CI)** | ***P* value** | **HR (95% CI)** | ***P* value** | **HR (95% CI)** | ***P* value** |
| **Total DAC including snack after dinner** | HR (95% CI) | P value | HR (95% CI) | P value | HR (95% CI) | P value |
| Continuous total DAC | 0.99 (0.96, 1.02) | 0.555 | 1.00 (0.95, 1.05) | 0.993 | 0.98 (0.94, 1.02) | 0.319 |
| Q1 (<2.45) | 1.00 (Reference) |  | 1.00 (Reference) |  | 1.00 (Reference) |  |
| Q2 (2.45 to 3.76) | 0.92 (0.76, 1.12) |  | 1.03 (0.71, 1.48) |  | 0.86 (0.70, 1.07) |  |
| Q3 (3.76 to 5.83) | 0.81 (0.65, 1.01) |  | 0.92 (0.63, 1.36) |  | 0.75 (0.58, 0.96) |  |
| Q4 (>5.83) | 0.88 (0.70, 1.09) | 0.129* | 0.95 (0.64, 1.40) | 0.668* | 0.82 (0.63, 1.06) | 0.087* |
| **Dinner DAC including snack after dinner** |  |  |  |  |  |  |
| Continuous dinner DAC | 0.99 (0.94, 1.04) | 0.638 | 1.01 (0.96, 1.05) | 0.806 | 0.96 (0.90, 1.03) | 0.233 |
| Q1 (<0.42) | 1.00 (Reference) |  | 1.00 (Reference) |  | 1.00 (Reference) |  |
| Q2 (0.42 to 0.91) | 0.97 (0.78, 1.20) |  | 0.81 (0.54, 1.20) |  | 1.05 (0.82, 1.34) |  |
| Q3 (0.91 to 1.91) | 0.88 (0.70, 1.11) |  | 0.81 (0.55, 1.19) |  | 0.91 (0.70, 1.19) |  |
| Q4 (>1.91) | 0.91 (0.75, 1.10) | 0.218* | 1.04 (0.76, 1.43) | 0.816* | 0.84 (0.66, 1.07) | 0.086* |
| **DAC in snack after dinner** |  |  |  |  |  |  |
| Continuous DAC in snack after dinner | 1.00 (0.94, 1.07) | 0.906 | 0.96 (0.82, 1.12) | 0.577 | 1.03 (0.96, 1.10) | 0.434 |
| Q1 (=0.00) | 1.00 (Reference) |  | 1.00 (Reference) |  | 1.00 (Reference) |  |
| Q2 (0.00 to 0.10) | 1.08 (0.85, 1.37) |  | 1.19 (0.81, 1.75) |  | 1.02 (0.78, 1.34) |  |
| Q3 (0.10 to 0.22) | 1.16 (0.90, 1.49) |  | 1.07 (0.67, 1.70) |  | 1.19 (0.91, 1.57) |  |
| Q4 (>0.22) | 1.23 (0.96, 1.57) | 0.077* | 1.06 (0.67, 1.65) | 0.933* | 1.30 (0.97, 1.75) | 0.031* |

Abbreviations: HR, hazard ratio; CI, confidence intervals; DAC, dietary total antioxidant capacity; HEI-2015, Healthy Eating Index 2015; BMI, body mass index; METs, metabolic equivalent score; CVD, cardiovascular disease. HR with 95%CI was assessed by weighted Cox regression analyses. Model was adjusted for, age, sex, and race, education, family income, dietary energy intake, alcohol consumption per day, smoke status, METs, BMI, serum HDL-Cholesterol, serum triglycerides, serum glycohemoglobin, diabetes, hypertension, CVD, dietary antioxidant supplement intake (vitamin C or vitamin E), and adherence to HEI-2015 score. Models for Total DAC including snack after dinner, dinner DAC including snack after dinner, and DAC in snack after dinner were further adjusted except the one that defined the group.
